# A Sleep‐Specific Midbrain Target for Sevoflurane Anesthesia

**DOI:** 10.1002/advs.202300189

**Published:** 2023-03-24

**Authors:** Tingting Yi, Na Wang, Jing Huang, Yaling Wang, Shuancheng Ren, Yiwen Hu, Jianxia Xia, Yixiang Liao, Xin Li, Fenlan Luo, Qin Ouyang, Yu Li, Ziyi Zheng, Qin Xiao, Rong Ren, Zhongxiang Yao, Xiangdong Tang, Yanjiang Wang, Xiaowei Chen, Chao He, Hong Li, Zhian Hu

**Affiliations:** ^1^ Department of Anesthesiology Second Affiliated Hospital Third Military Medical University Chongqing 400037 China; ^2^ Department of Physiology Third Military Medical University Chongqing 400038 China; ^3^ College of Bioengineering Chongqing University Chongqing 400044 China; ^4^ School of Pharmacy Third Military Medical University Chongqing 400038 China; ^5^ Sleep Medicine Center Department of Respiratory and Critical Care Medicine Mental Health Center West China Hospital Sichuan University Chengdu 610041 China; ^6^ Department of Neurology Daping Hospital Third Military Medical University Chongqing 400042 China; ^7^ Brain Research Center Collaborative Innovation Center for Brain Science Third Military Medical University Chongqing 400038 China; ^8^ Department of Anesthesiology Yongchuan Hospital Chongqing Medical University Chongqing 402160 China; ^9^ Chongqing Institute for Brain and Intelligence Guangyang Bay Laboratory Chongqing 400064 China

**Keywords:** Edinger‐Westphal nucleus, general anesthesia, growth hormone secretagogue receptor, sevoflurane, sleep

## Abstract

Sevoflurane has been the most widely used inhaled anesthetics with a favorable recovery profile; however, the precise mechanisms underlying its anesthetic action are still not completely understood. Here the authors show that sevoflurane activates a cluster of urocortin 1 (UCN1^+^)/cocaine‐ and amphetamine‐regulated transcript (CART^+^) neurons in the midbrain involved in its anesthesia. Furthermore, growth hormone secretagogue receptor (GHSR) is highly enriched in sevoflurane‐activated UCN1^+^/CART^+^ cells and is necessary for sleep induction. Blockade of GHSR abolishes the excitatory effect of sevoflurane on UCN1^+^/CART^+^ neurons and attenuates its anesthetic effect. Collectively, their data suggest that anesthetic action of sevoflurane necessitates the GHSR activation in midbrain UCN1^+^/CART^+^ neurons, which provides a novel target including the nucleus and receptor in the field of anesthesia.

## Graphical abstract

Sevoflurane is one of the most widely used inhaled anesthetics; however, the mechanisms underlying its anesthetic action are still not completely understood. This work identifies a cluster of sleep‐related urocortin 1 (UCN1^+^)/cocaine‐ and amphetamine‐regulated transcript (CART^+^) neurons in the midbrain with high enrichment of the growth hormone secretagogue receptor (GHSR). Sevoflurane activates the UCN1^+^/CART^+^ neurons through GHSR to promote anesthesia.

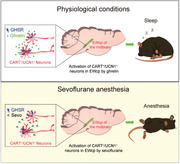

## 1 Introduction

Inhaled anesthetics, which were first used a century and a half ago, are of great significance to modern medicine.^[^ ^1^, ^2^ ^]^ As the most widely used inhaled anesthetics, sevoflurane is characterized by its low blood‐gas solubility, hemodynamic stability, and favorable recovery profile.^[^ ^3^, ^4^, ^5^ ^]^ For several decades, a great deal of effort has gone into identifying the targets underlying the effects of general anesthetics including sevoflurane. Many reports have shown that sevoflurane works directly through exerting a wide range of inhibitory impacts on neocortical regions, such as frontal and parietal cortices.^[^ ^5^, ^6^, ^7^, ^8^, ^9^ ^]^ It is widely considered that these inhibitory impacts might be mainly due to the fact that sevoflurane could potentiate *γ*‐aminobutyric acid type A receptors (GABA_A_Rs) and thus enhance inhibitory inputs.^[^ ^6^, ^10^, ^11^ ^]^

Despite the pioneering work, the cellular and molecular mechanisms underlying anesthetic action of sevoflurane are still not completely understood. It should be noted that the brain states are controlled by the subcortical wakefulness‐ and sleep‐promoting nuclei. The activation of the neurons in the sleep‐promoting nuclei, such as the ventrolateral preoptic nucleus, supraoptic nucleus (SON), and parafacial zone, could suppress the wakefulness‐promoting nuclei and then promote the transitions from wakefulfulness to sleep. Although there are many differences between sleep and general anesthesia, mounting evidence indicates they share some common features, such as unconsciousness and amnesia, and anesthetics could work by affecting the sleep/wakefulness‐promoting nuclei.^[^ ^12^, ^13^, ^14^ ^]^ For instance, it has been reported that sevoflurane could inhibit the wakefulness‐promoting neurons, including dopamine D1 receptor‐positive neurons in the nucleus accumbens,^[^ ^7^ ^]^ orexinergic neurons in the hypothalamus,^[^ ^8^ ^]^ medial parabrachial neurons^[^ ^6^ ^]^ and the paraventricular thalamic neurons projecting to the bed nucleus of the stria terminalis (BNST)^[^ ^15^ ^]^ and contribute to its anesthetic action.

Notably, a few recent studies have begun to highlight the excitatory effects on these sleep‐promoting nuclei exerted by general anesthetics. It has been reported that isoflurane, dexmedetomidine, ketamine, and propofol activate sleep‐related neurons of the SON to stabilize anesthesia.^[^ ^13^ ^]^ Dexmedetomidine increases the number of c‐fos^+^ neurons in the preoptic area to induce sedation and mimic natural sleep.^[^ ^16^ ^]^ However, it remains unresolved whether sevoflurane exerts its anesthetic effect by exciting yet unidentified neurons in the brain.

In this study, we discovered that sevoflurane activates a cluster of neurons in the Edinger‐Westphal (EW) nucleus of the midbrain. The EW nucleus subdivides into the preganglionic EW (EWpg) and the centrally projecting EW (EWcp) regions.^[^ ^17^, ^18^ ^]^ The EWpg is part of the oculomotor nuclear complex and is previously known for its control of the ciliary reflex.^[^ ^19^ ^]^ EWcp contains multiple types of neurons including urocortin 1 (UCN1^+^)/cocaine‐ and amphetamine‐regulated transcript (CART^+^) peptidergic, dopaminergic, and glutamatergic neurons.^[^ ^20^, ^21^, ^22^, ^23^, ^24^ ^]^ Behavioral evidence suggests that EWcp is related to addiction, attention, maternal preparatory nesting, stress responses, and fear modulation,^[^ ^22^, ^23^, ^24^, ^25^, ^26^, ^27^ ^]^ but a definitive role for these different cell types remains elusive. Here we show that ghrelin/growth hormone secretagogue receptor (GHSR) activation in the EWcp UCN1^+^/CART^+^ neurons is involved in sleep induction and anesthetic action of sevoflurane. These results provide a novel target including the nucleus and receptor in the field of anesthesia.

## 2 Results

### 2.1 Sevoflurane Activates the Sleep‐Related Midbrain Neurons

**Figure 1:**
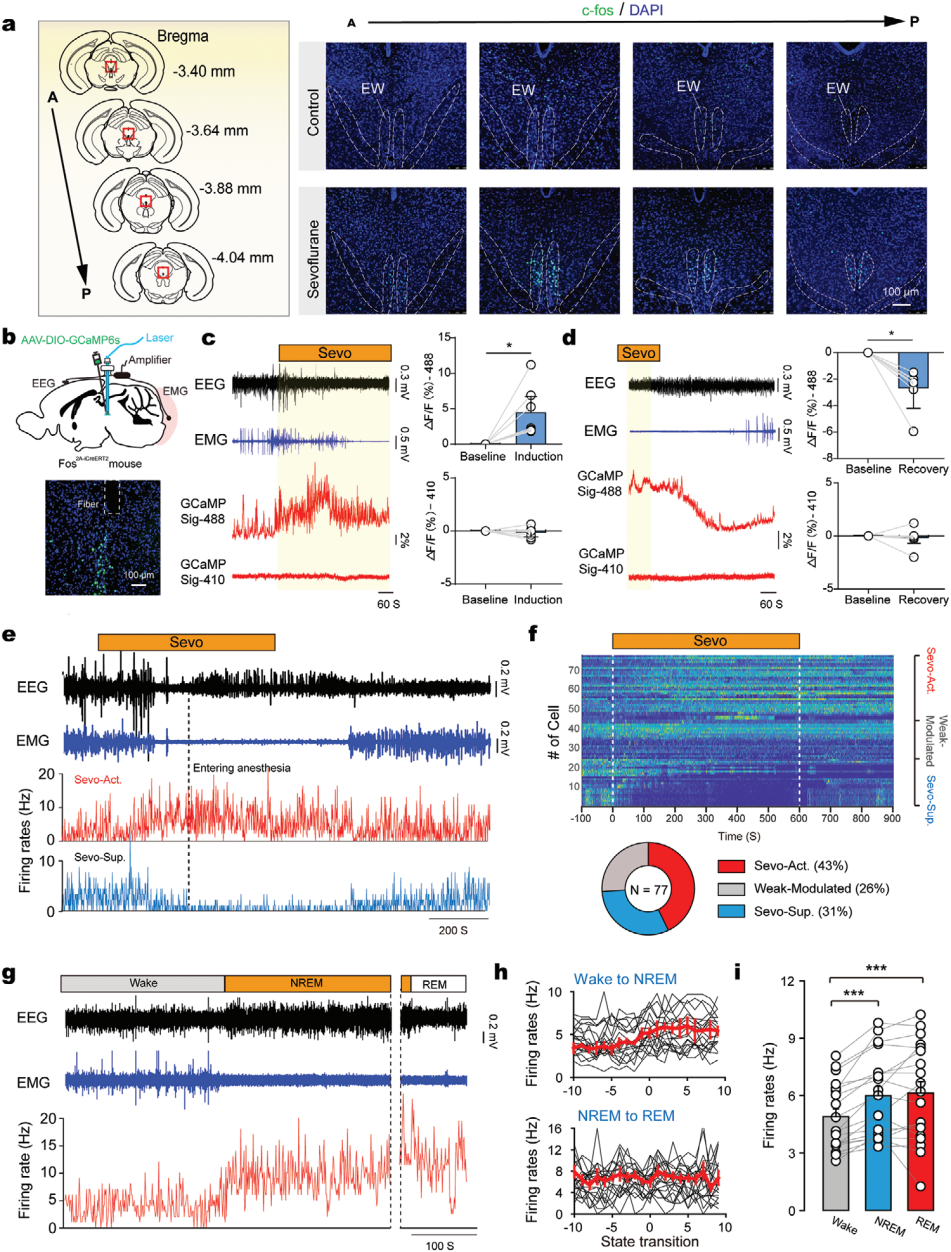
Sevoflurane activates the midbrain neurons that are related to sleep. a) The cluster of cells in midbrain Edinger Westphal (EW) nucleus was activated by sevoflurane. A, anterior. P, posterior. b) Top, schematic of AAV2/9‐Ef1a‐DIO‐jGCaMP6s injected into the EW of Fos^2A‐iCreERT2^ mice. Bottom, GCaMP6s/DAPI immunofluorescence in EW neurons and track of the optic fiber implanted above the EW. c) Left, representative traces of electroencephalogram (EEG), electromyography (EMG), Ca^2+^ signals before and during 2% sevoflurane exposure. Right, quantification of Ca^2+^ signal changes during baseline and induction (mean ± SEM). Wilcoxon matched‐pairs signed rank test, * *P* < 0.05, *n* = 7 mice. d) Left, representative traces of EEG, EMG, Ca^2+^ signals during and after 2% sevoflurane exposure. Right, quantification of Ca^2+^ signal changes during baseline and recovery (mean ± SEM). Wilcoxon matched‐pairs signed rank test, * *P* < 0.05, *n* = 7 mice. e) Representative traces of EEG and EMG, the sevoflurane‐suppressed (Sevo‐Sup.) and sevoflurane‐activated (Sevo‐Act.) neurons. Black dashed lines indicate the time point of entering anesthetic state. f) Top, activity profile of all neurons recorded. The spike rate of each neuron was normalized by its peak firing rate. Bottom, classification of the neuronal population based on its response toward sevoflurane. g) The firing rate of an example sevoflurane‐activated neuron recorded during wakefulness, nonrapid eye movement sleep (NREM) sleep and rapid eye movement sleep (REM) sleep. h) Firing rates of sevoflurane‐activated neurons (*n* = 19) during state transitions: wake‐to‐NREM (top) and REM‐to‐NREM (bottom). i) Histograms showing the average firing rates of sevoflurane‐activated neurons during wake, NREM, and REM states (mean ± SEM). Friedman repeated measures analysis of variance on ranks, *** *P* < 0.001, *n* = 19 neurons.

We visualized a brain‐wide map of c‐fos expression after a period of sevoflurane (2%) anesthesia or oxygen exposure alone for 2 h in C57BL/6 mice. Sevoflurane did not significantly increase the number of c‐fos^+^ neurons in cortical areas, such as primary sensory and motor cortices, or classic subcortical wakefulness/sleep‐related brain areas, such as paraventricular nucleus of thalamus, reticular nucleus of thalamus, or dorsal raphe nucleus^[^ ^28^, ^29^, ^30^ ^]^ (Figure S1, Supporting Information). However, sevoflurane anesthesia increased c‐fos^+^ immunoreactivity in several other regions including the SON, central amygdala, and the oval division of the bed nucleus of the stria terminalis (ovBNST) (Figure S1, Supporting Information), known to be activated by other anesthetics and related to sleep and analgesia.^[^ ^13^, ^31^ ^]^ Moreover, we observed a distinct cluster of c‐fos^+^ neurons mainly located in the EWcp of the midbrain (from −2.80 to −4.2 mm behind the bregma) (**Figure** **1**a).

Then, we wanted to know the dynamic activity pattern of sevoflurane‐activated EWcp cells in vivo. We first utilized the targeted recombination in active populations (TRAP) technology to specifically label sevoflurane‐activated neurons, using a Fos^2A‐iCreERT2^ transgenic mouse line that expressed tamoxifen‐inducible improved Cre recombinase from the *Fos* promoter/enhancer elements. We injected AAV2/9‐Ef1a‐DIO‐jGCaMP6s into the EWcp region in Fos^2A‐iCreERT2^ mice, followed by a 5‐day continuous intraperitoneal injection of tamoxifen starting 1 week after the virus injection. These mice were then exposed to sevoflurane (2%) anesthesia for 2 h to make the sevoflurane‐activated neurons specifically express GCaMP6s (Figure 1b; Figure S2a,b, Supporting Information). The fiber photometry was applied to detect Ca^2+^ activities of sevoflurane‐activated neurons with simultaneous monitoring of electroencephalogram (EEG) and electromyography (EMG). Interestingly, the Ca^2+^ activities of sevoflurane‐activated neurons began to increase gradually after sevoflurane administration. When entering the anesthesia determined by the occurrence of slow wave power in EEG and minimal activity in EMG,^[^ ^13^ ^]^ the Ca^2+^ activities of sevoflurane‐activated neurons reach a high level (Figure 1c). Furthermore, some studies reported that the onset of burst suppression on the EEG was used to assess whether the subjects enter the unconscious and deep anesthetic state.^[^ ^7^, ^32^, ^33^ ^]^ We further analyzed the data of photometry with this criteria. Consistently, we found that the activity of sevoflurane‐activated neurons significantly increased during deep anesthetic state compared with baseline. In contrast, after the cease of sevoflurane administration, Ca^2+^ activities began to decrease (Figure 1d; Figure S2c, Supporting Information). These results further suggest that sevoflurane could activate a cluster of the neurons in the EWcp region.

In addition, we placed an electrode array in the sevoflurane‐activated EWcp region for multiple‐channel single‐unit recordings combined with simultaneous monitoring of EEG and EMG (Figure S2d, Supporting Information). In total, we recorded 77 cells (*n* = 16 mice), of which 43% showed an increase in their discharge frequency during sevoflurane (2%) anesthesia, 26% remained unchanged, and 31% showed a decrease (Figure 1e,f). Interestingly, among these neurons activated by sevoflurane, the discharge frequency of almost all cells (30/33, 91%) began to increase gradually after sevoflurane administration. When entering the anesthesia state, the discharge frequency peaked (Figure S2e, Supporting Information). This kinetic characteristic suggests that sevoflurane‐activated cells begin to function while entering anesthesia, rather than indirectly changing their activity after entering anesthesia.

Among sevoflurane‐activated cells, we further detected the changes in the discharge frequency of 19 cells (*n* = 10 mice) across sleep‐wake cycle under physiological condition (Figure 1g). Interestingly, the firing rate of all these sevoflurane‐activated cells gradually increased as non‐rapid eye movement (NREM) sleep was approaching. However, no difference was observed during transitions from NREM to rapid eye movement (REM) sleep (Figure 1h). On average, these sevoflurane‐activated cells showed the highest level of discharge frequency during both NREM and REM sleep, and the lowest level of discharge frequency during wakefulness (Figure 1i). These data suggest that sevoflurane activates sleep‐related midbrain neurons.

### 2.2 Sevoflurane‐Activated Midbrain Neurons Participate in its Anesthetic Action

**Figure 2:**
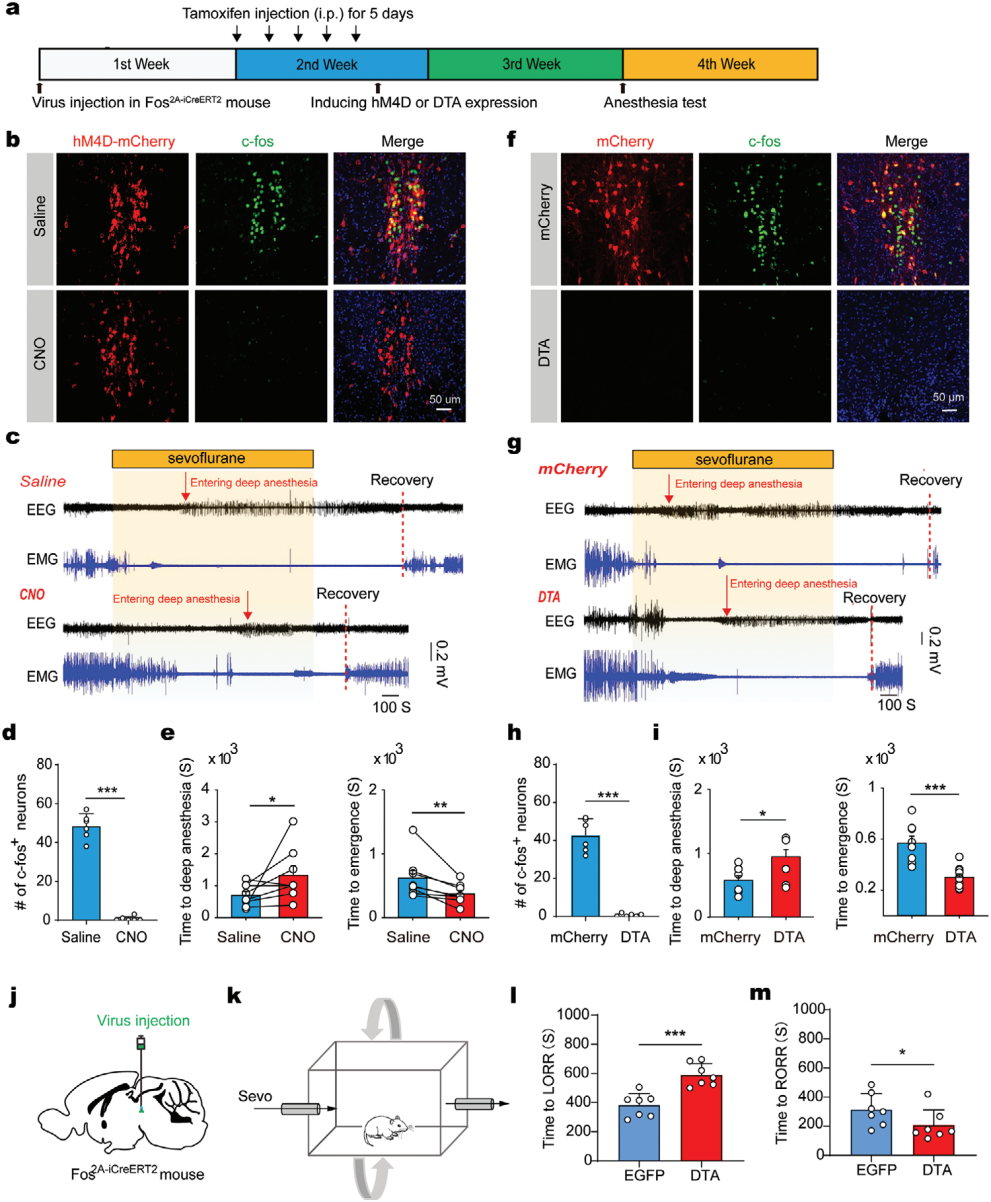
Sevoflurane‐activated midbrain neurons participate in sevoflurane anesthesia. a) Schematic diagram of TRAP technology to specifically manipulate the sevoflurane‐activated neurons. b) Representative immunofluorescence (IF) images of TRAP‐captured sevoflurane‐activated neurons (red) and c‐fos^+^ neurons (green) induced by re‐exposure to sevoflurane. c) Representative traces of EEG and EMG before, during, and after application of sevoflurane in saline and CNO groups. d) The number of c‐fos^+^ neurons expressing hM4D‐mCherry in the midbrain EW region after injection of saline or CNO (mean ± SEM). Unpaired *t*‐test, *** *P* < 0.001, *n* = 6 sections from 3 mice. e) Left, time to anesthesia with sevoflurane exposure after intraperitoneal (i.p.) injection of saline or CNO (mean ± SEM). Wilcoxon signed rank test, *P < 0.05, *n* = 8 mice for each group. Right, emergence time with sevoflurane exposure after i.p. injection of saline or CNO (mean ± SEM). Wilcoxon signed rank test, **P < 0.01, *n* = 8 mice for each group. f) Representative IF images of TRAP‐captured sevoflurane‐activated neurons (red) and c‐fos^+^ neurons (green) induced by re‐exposure to sevoflurane in the mCherry and DTA groups. g) Representative traces of EEG and EMG before, during, and after administration of sevoflurane in the mCherry and DTA groups. h) The histograms showing the number of c‐fos^+^ neurons in the midbrain EW region in mCherry and DTA groups (mean ± SEM). Unpaired *t*‐test, *** *P* < 0.001, *n* = 6 sections from 3 mice. i) Left, time to deep anesthesia with sevoflurane exposure in the mCherry and DTA groups (mean ± SEM). Unpaired mann‐whitney rank sum test, *P < 0.05, mCherry: *n* = 8 mice; DTA: *n* = 10 mice. Right, anesthetic emergence time with sevoflurane exposure in the mCherry and DTA groups (mean ± SEM). Unpaired t test, ***P < 0.001, mCherry: *n* = 8 mice; DTA: *n* = 10 mice. j) Schematic diagram of virus injection. k) Experimental setup for assessing loss of right reflex in the presence of continuous sevoflurane. l) The time to the loss of righting reflex with sevoflurane exposure (mean ± SEM). Unpaired test, ***P < 0.001, *n* = 7 mice. m) The time to the recovery of righting reflex with sevoflurane exposure (mean ± SEM). Mann Whitney test, *P < 0.05, *n* = 7 mice. LORR, loss of righting reflex. RORR, recovery of righting reflex.

The anesthetic state and natural sleep manifest some common physiological traits, such as unconsciousness, amnesia, and reduced locomotor activity. If sevoflurane‐activated EWcp neurons were sleep‐related, we speculated that these cells would be involved in sevoflurane anesthesia. To test this hypothesis, we utilized the TRAP technology to specifically inhibit sevoflurane‐activated neurons. We first injected AAV2/9‐hSyn‐DIO‐hM4D(Gi)‐mCherry into the EWcp region in Fos^2A‐iCreERT2^ mice, followed by a 5‐day continuous intraperitoneal injection of tamoxifen starting 1 week after the virus injection. These mice were then exposed to sevoflurane (2%) anesthesia for 2 h to label the sevoflurane‐activated neurons with hM4D‐mCherry. We found that ≈80% of TRAP‐labeled neurons were c‐fos positive (Figure S3a, Supporting Information), suggesting that TRAP is specific for labeling sevoflurane‐activated neurons. As it takes tens of minutes to effectively inhibit these cells during anesthesia, we selected chemogenetic approach, but not optogenetic stimulation, which would produce nonspecific effects such as tissue heating or photodamage due to long‐term light stimulation (**Figure** **2**a,b). Whole‐cell patch‐clamp recordings of hM4D‐mCherry positive neurons from brain slices confirmed that clozapine‐N‐oxide (CNO) potently inhibited these neurons (Figure S3b, Supporting Information). Furthermore, there was almost no c‐fos expression in the sevoflurane‐activated midbrain region after CNO administration (Figure 2b,d).

It has been reported that EEG delta (0.5‐4 Hz) power gradually increases from awakening to anesthesia. Upon reaching anesthesia, burst suppression continuously occurs.^[^ ^7^ ^]^ In order to determine the time to anesthesia objectively, we analyzed the EEG power spectrum, and an exponential function was used to fit the ratio of EEG theta (4–12 Hz) to delta wave power (theta/delta ratio) against time. We took the time point when the theta/delta ratio decreased to that of the burst suppression as the time point of entrance into anesthesia (Figure S4a,b, Supporting Information). Under this condition, we found that inhibition of sevoflurane‐activated neurons by systemic CNO treatment caused a higher theta/delta ratio during induction of anesthesia and significantly prolonged the time to anesthesia when exposed to sevoflurane (Figure 2c,e and Figure S4c, Supporting Information), in comparison to saline treatment. In addition, we also detected the emergence time of mice after sevoflurane cessation. The animals showed high‐frequency EEG and continuous high‐amplitude EMG at the emergence time point. Consistently, the emergence time was accelerated after administration of CNO (Figure 2c,e).

In the wild‐type control mice or the control mice with EW sevoflurane‐activated neurons only expressing mCherry, CNO administration did not affect the anesthetic induction and emergence time (Figure S5, Supporting Information). Altogether, these results indicate that CNO administration‐induced selective inhibition of sevoflurane‐activated neurons, but not CNO administration itself, decreases sevoflurane sensitivity.

To further confirm these findings, we next determined whether ablation of sevoflurane‐activated neurons will affect sevoflurane anesthesia. We injected AAV2/9‐Ef1a‐DIO‐DTA mixed with AAV2/9‐hSyn‐DIO‐mCherry, or control AAV2/9‐hSyn‐DIO‐mCherry virus, into EWcp to specifically ablate sevoflurane‐activated neurons using TRAP technology (Figure 2a). The DTA‐mediated ablating effect was confirmed by c‐fos staining. In control mice, we observed TRAP neurons expressing both mCherry and c‐fos, while almost no c‐fos expression was detected in DTA‐mediated ablation mice (Figure 2f,h). Additionally, immunohistochemical staining showed that the expression level of the NeuN^+^, a widely used neuronal marker, was significantly decreased in the sevoflurane‐activated EWcp after DTA‐mediated ablation as compared to the control, indicating the effectiveness of the DTA‐mediated ablation (Figure S6a, Supporting Information). To further validate the specificity of the ablation approach, we used immunofluorescence (IF) and fluorescence in situ hybridization (FISH) to detect the other cell types including glutamatergic, GABAergic, dopaminergic, and substance P neurons that have been detected around the sevoflurane‐activated EWcp of the midbrain.^[^ ^22^, ^23^ ^]^ Indeed, DTA only ablates sevoflurane‐activated c‐fos^+^ neurons without effects on the number of other types of cells, demonstrating the specificity of the DTA‐medaited ablation (Figures S6,b–f, Supporting Information).

By precisely analyzing the EEG power spectrum, ablation of sevoflurane‐activated neurons significantly increased the theta/delta power ratio during the induction of anesthesia and delayed the time to anesthesia (Figure 2g,i and Figure S4d, Supporting Information). In addition, the time to recovery from sevoflurane anesthesia was also significantly shortened after DTA‐mediated ablation of sevoflurane‐activated neurons (Figure 2i).

To further validate whether sevoflurane‐activated neurons are important targets for sevoflurane anesthesia, we carried out behavioral research and tested the changes in righting reflex during sevoflurane anesthesia after DTA‐mediated ablation of sevoflurane‐activated neurons. Consistently, we observed that ablation of sevoflurane‐activated midbrain cells indeed delayed induction of and accelerated emergence from anesthesia (Figure 2, j–m). In sum, these findings suggest that these sevoflurane‐activated neurons in the midbrain are an important component for sevoflurane action. EWcp cells mainly project to the hypothalamus, the zona incerta, the dorsal raphe, the ventrolateral periaqueductal gray, and the brainstem reticular formation.^[^ ^23^, ^34^ ^]^ These downstream brain regions of the EWcp cells, such as hypothalamus, the dorsal raphe, and the ventrolateral periaqueductal gray known to be involved in sleep/arousal control,^[^ ^13^, ^16^, ^29^ ^]^ might be the important basis for sevoflurane action.

### 2.3 Sevoflurane‐Activated Midbrain Neurons are GHSR‐Enriched UCN1^+^/CART^+^ Neurons that are Required for Sleep Induction

**Figure 3:**
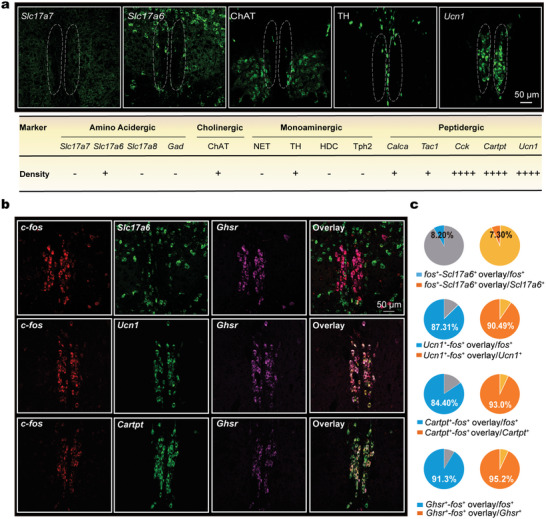
Sevoflurane‐activated UCN1^+^/CART^+^ neurons are highly enriched for growth hormone secretagogue receptor (GHSR). a) The cell types in the midbrain area activated by sevoflurane were detected using IF and fluorescence in situ hybridization (FISH). Expressions of NET, ChAT, TH, HDC, and Tph2 were detected using IF, and the remaining markers were detected by FISH. *n* = 3 mice. b) Representative images of three‐color in situ hybridization among *c‐fos* (red) marking sevoflurane‐activated neurons, *Ghsr* (purple), and the following probes (green): *Slc17a6* (encoding VGLUT2)*, Ucn1and Cartpt*. *n* = 3 mice. c) Pie chart of the percentage of sevoflurane‐activated neurons (*c‐fos* ^+^) colocalized with each probe.

Next, we identified the cell types in the midbrain area activated by sevoflurane. We first used IF and FISH to explore the cell type spectrum in the EW region. We detected markers of glutamatergic neurons and GABAergic neurons along the sevoflurane‐activated midbrain region. We found that vesicular glutamate transporter 2 (VGLUT2, encoded by *Slc17a6*), but not VGLUT1 (encoded by *Slc17a7*), VGLUT3 (encoded by *Slc17a8*) or glutamic acid decarboxylase (GAD), was expressed in the EWcp region. Additionally, we observed immunoreactivity to acetyltransferase (ChAT) and tyrosine hydroxylase (TH), markers for cholinergic and dopaminergic neurons respectively, but could not detect signals corresponding to noradrenergic, histaminergic, or serotonergic neurons (**Figure** **3**a and Figure S7, Supporting Information). It has been reported that neurons in the midbrain EW area express many peptides, such as calcitonin gene‐related polypeptide‐alpha (CALCA), substance P neuron marker tachykinin 1 (TAC1), cholecystokinin (CCK), CART and UCN1 peptides. We further confirmed by FISH that these peptides were indeed expressed in this region (Figure 3a and Figure S7, Supporting Information). In sum, these results suggest that multiple types of cells including glutamatergic, GABAergic, cholinergic, dopaminergic, CALCA^+^, substance P, CCK^+^, and UCN1^+^ neurons exist in the midbrain area activated by sevoflurane.

Sevoflurane‐activated c‐fos^+^ neurons did not express ChAT or TH. None of these *c‐fos* ^+^ neurons expressed the GABAergic marker *Gad*, substance P neuron marker *Tac1*, or *Calca* (Figure S8, Supporting Information), a marker for a subtype of glutamatergic neurons that have been reported to be involved in sleep generation.^[^ ^35^ ^]^ Only a very small proportion of sevoflurane‐activated neurons were glutamatergic cells (Figure 3b,c and Figure S9, Supporting Information). Interestingly, the overlap between *Cartpt* (encoding CART) and *c‐fos*, and between *Ucn1* and *c‐fos* mRNA expression reached nearly 90% (Figure 3b,c). Further, FISH showed a complete overlap of *Cartpt* and *Ucn1* labeling (> 98%) (Figure S10a, Supporting Information). UCN1^+^ neurons have also been reported to be mainly CCK‐positive.^[^ ^22^, ^26^ ^]^ None of the UCN1^+^ neurons expressed VGLUT2 or vesicular GABA transporter (VGAT, encoded by *Slc32a1*) (Figure S10b,c, Supporting Information). These results suggest that sevoflurane‐activated neurons are mainly UCN1^+^ and CART^+^ neurons, but not glutamatergic or GABAergic neurons. We therefore named sevoflurane‐activated cells UCN1^+^/CART^+^ neurons in the following experiments.

In situ hybridization map of the Allen Mouse Brain Atlas and other previous studies have shown the high expression of growth hormone secretagogue receptors (GHSR) mRNA in the EW region.^[^ ^36^ ^]^ Consistent with these previous findings, we indeed observed high expression levels of GHSR in the sevoflurane‐activated midbrain. Interestingly, FISH showed that GHSR was mainly (95%) colocalized with sevoflurane‐activated c‐fos^+^ and UCN1^+^/CART^+^ neurons, but not glutamatergic neurons (Figure 3b,c).

**Figure 4:**
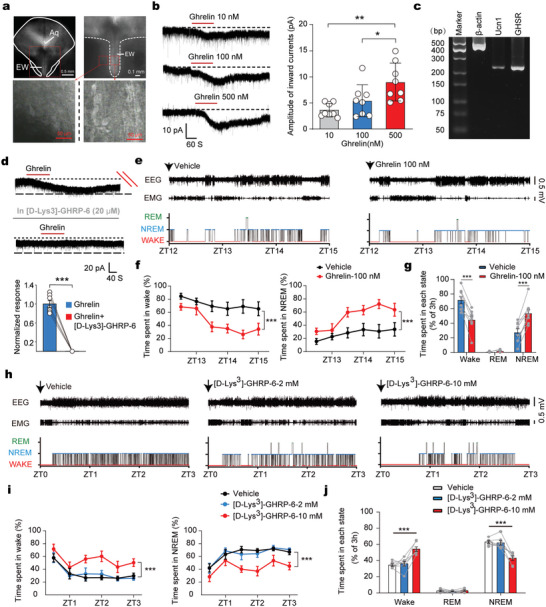
GHSR activation in the sevoflurane‐activated UCN1^+^/CART^+^ neurons is indispensable for sleep induction. a) Representative images showing the recording regions in the sevoflurane‐activated midbrain EW regions. EW region exhibited a light zone, while the outside of EW showed a dark zone. b) Whole‐cell patch‐clamp recording of EW neurons following treatments of different concentrations of ghrelin peptide, an agonist for GHSR, noting that ghrelin generated inward currents in a concentration‐dependent manner (mean ± SEM). One way analysis of variance, *P < 0.05, **P < 0.01, *n* = 8 cells. c) scRT‐PCR combined with whole‐cell patch clamp showed that the neurons (*n* = 11 independent cells) displayed inward currents in response to ghrelin were positive for Ucn1 and GHSR. d) Effect of ghrelin (100 nM) on membrane currents before and during application of GHSR antagonist (mean ± SEM). Paired *t*‐test, ***P < 0.001, *n* = 7 cells. e) Representative traces of EEG, EMG, and hypnogram during 3 h postvehicle or ghrelin (100 nM) injection into midbrain EW region during the dark phase. f) Left, hourly percentage of time spent in wakefulness state post vehicle or ghrelin (100 nM) injection. Right, hourly percentage of time spent in nonrapid eye movement sleep (NREM) state during 3 h postvehicle or ghrelin (100 nM) injection. g) Representative traces of EEG, EMG, and hypnogram during 3 h postvehicle or ghrelin (100 nM) injection into the midbrain EW region during the dark phase (mean ± SEM). Paired *t*‐test, ***P < 0.001, *n* = 8 mice. h) Representative traces of EEG, EMG, and hypnogram during 3 h post vehicle or different concentrations of GHSR antagonist injection into midbrain EW region during the light phase. i) Hourly percentage of time spent in wakefulness (left) and NREM (right) state post vehicle or GHSR antagonist injection. j) Percentage of time spent in each state during 3 h post vehicle or GHSR antagonist injection (mean ± SEM). One‐way repeated measures analysis of variance, ***P < 0.001, *n* = 8 mice.

Ghrelin peptide, an agonist for GHSR, has been reported to promote slow‐wave sleep in humans.^[^ ^37^, ^38^, ^39^ ^]^ Additionally, sevoflurane‐activated UCN1^+/^CART^+^ cells are active during sleep. Based on these clues, we hypothesized that ghrelin/GHSR signaling might excite the UCN1^+^/CART^+^ neurons and is required for sleep induction under physiological conditions. We used whole‐cell patch‐clamp to record neurons in the sevoflurane‐activated midbrain regions. Midbrain EWcp and the surrounding region could be clearly distinguished in acute brain slices. The EWcp midbrain region showed strong light transmittance in the acute brain slice and was therefore named light zone, while the surrounding region displayed weak light transmittance and was darker than EWcp region (**Figure** **4**a). For all recorded cells, bath application of the ghrelin peptide (10–500 nM) indeed dose‐dependently generated inward currents (Figure 4b). The single‐cell reverse transcription‐polymerase chain reaction (scRT‐PCR) combined with whole‐cell recoding showed that all the neurons (*n* = 11) displaying inward currents in response to ghrelin peptide were positive for both UCN1 and GHSR (Figure 4c). The ghrelin‐induced inward currents were blocked by a GHSR antagonist (Figure 4d), indicating that GHSR activation could excite the UCN1^+^/CART^+^ neurons.

We also determined whether the action of ghrelin was a direct postsynaptic action. Ghrelin peptide‐induced inward currents persisted after blockade of the inhibitory and excitatory synaptic transmissions, suggesting a direct postsynaptic excitatory response (Figure S11a, Supporting Information). Under the current‐clamp mode, ghrelin (10 nM) strongly depolarized the membrane potentials of the UCN1^+^/CART^+^ neurons and increased the firing frequency of these recorded neurons (Figure S11b, Supporting Information).

Then, we locally injected ghrelin or saline at the beginning of the dark phase (18:00, ZT12), when mice spend most of their time awake. Initially, we locally injected ghrelin at a low concentration (10 nM). Under this condition, sleep‐wake behavior was unaffected (Figure S12, Supporting Information). When the concentration of ghrelin was increased to 100 nM, we observed a significant increase in NREM sleep compared to saline controls (Figure 4e–g).

Next, we explored whether endogenous ghrelin/GHSR signaling is involved in the occurrence of sleep under physiological conditions. We locally injected GHSR antagonist or saline at the beginning of the light phase (6:00, ZT0), when mice spend most of their time asleep. Local infusion of GHSR antagonist in the midbrain EW region at low concentration (2 mM) did not affect the time spent in either NREM or REM sleep. However, when the concentration of GHSR antagonist reached 10 mM, we observed a significant increase in wakefulness (Figure 4h–j). These findings suggest that the excitatory effect of ghrelin/GHSR signaling on UCN1^+^/CART^+^ neurons could control sleep induction under physiological condition.

### 2.4 Anesthetic Action of Sevoflurane Necessitates GHSR Activation in UCN1^+^/CART^+^ Neurons

Although important differences exist between sleep and anesthetized state, the similarities have led to speculation that natural sleep and anesthetic state might share common neural substrates.^[^ ^12^, ^13^, ^16^ ^]^ Thus, we wanted to know whether the sleep‐promoting ghrelin/GHSR signaling in the UCN1^+^/CART^+^ neurons is involved in sevoflurane anesthesia. Similar to the effect of ghrelin, bath application of 0.03%–0.4% sevoflurane, the concentration was clinically relevant based on previous studies,^[^ ^40^, ^41^ ^]^ also dose‐dependently induced inward currents in the EWcp region (Figure S13a, Supporting Information). Consistent with our morphological data, scRT‐PCR combined with whole‐cell patch‐clamp showed that all the neurons (*n* = 11) that exhibited inward currents in response to sevoflurane were positive for UCN1, but not VGLUT2 (Figure S13b, Supporting Information). Under current‐clamp mode, sevoflurane depolarized the membrane potentials and strongly increased the firing frequency of UCN1^+^/CART^+^ neurons (Figure S13c, Supporting Information). However, some other cells recorded in the dark zone had either no response, or outward current response, to sevoflurane. The scRT‐PCR showed that these neurons without inward current response were negative for UCN1, and a small proportion (36%) of these neurons were positive for VGLUT2 (Figure S13d,e, Supporting Information).

We further increased the number of recorded cells (*n* = 173) in and around the sevoflurane‐activated midbrain EWcp region to explore the spatial distribution of these cells with different responses. We found that cells with inward current response were mainly distributed in the midbrain EWcp region, which showed an increase in c‐fos expression after application of sevoflurane. On the contrary, cells without an inward response were mainly distributed outside the activated region (Figure S13f, Supporting Information).

Is the excitatory action of sevoflurane a direct or indirect effect caused by alteration of synaptic transmission? Application of sevoflurane onto EW UCN1^+^/CART^+^ neurons evoked a stable inward current instead of excitatory postsynaptic currents/inhibitory postsynaptic currents, suggesting a direct postsynaptic excitatory effect.^[^ ^42^, ^43^ ^]^ Additionally, we found that the sevoflurane‐induced inward current response in UCN1^+^/CART^+^ neurons was not affected after blockade of inhibitory and excitatory synaptic transmission (Figure S13g, Supporting Information). These results suggest that sevoflurane directly excites UCN1^+^/CART^+^ neurons independently of synaptic transmission.

**Figure 5:**
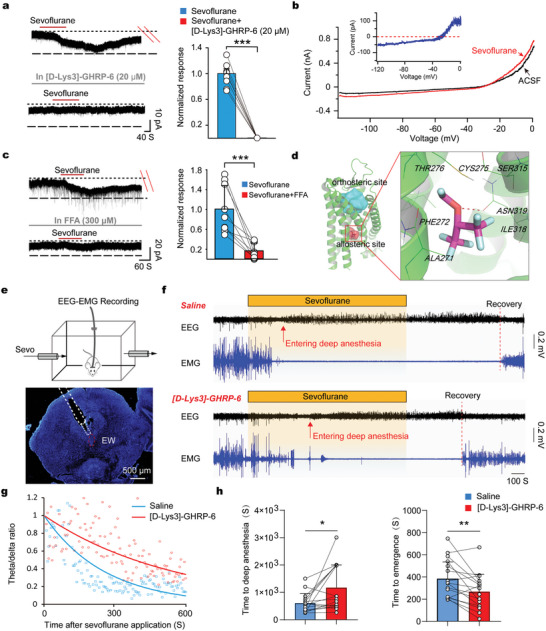
GHSR activation in the sevoflurane‐activated UCN1^+^/CART^+^ neurons contributes to sevoflurane anesthesia. a) Left, examples of raw traces showing the effects of sevoflurane on the membrane currents in the presence of GHSR antagonist. Right, histograms summarizing the changes of sevoflurane‐induced inward currents before and after blocking GHSR (mean ± SEM). Paired *t*‐test, ***P < 0.001, *n* = 7 cells. b) The current‐voltage curve in the absence or presence of sevoflurane. Inset, subtraction of the current prior to the application of sevoflurane generated a net current with a reversal potential of −30 mV. c) Left, examples of raw traces showing the effects of sevoflurane on the membrane currents in the presence of NSCC antagonist. Right, histograms summarizing the changes of sevoflurane‐induced inward currents before and after blocking NSCCs (mean ± SEM). Wilcoxon signed rank test, ***P < 0.001, *n* = 9 cells. d) The docking mode of sevoflurane/GHSR interaction. e) Top, experimental setup for EEG‐EMG recordings in the presence of continuous sevoflurane. Bottom, DAPI staining showing the implantation of drug cannula in the midbrain EW region. f) Representative traces of EEG and EMG before, during, and after application of sevoflurane in saline and GHSR antagonist groups. g) The EEG theta (4‐12 Hz)/delta (0.5–4 Hz) power ratio after application of sevoflurane in mice with local injection of saline and GHSR antagonist. The line plot represented the exponential function that was used to fit the theta/delta ratio. h) Left, time to deep anesthesia with sevoflurane exposure after local injection of saline or GHSR antagonist (mean ± SEM). Wilcoxon signed rank test, *P < 0.05, *n* = 16 mice. Right, emergence time with sevoflurane exposure after local injection of saline or GHSR antagonist (mean ± SEM). Paired *t*‐test, **P < 0.01, *n* = 16 mice.

As the GHSR activation could excite the UCN1^+^/CART^+^ neurons, we wanted to know whether sevoflurane activates the UCN1^+^/CART^+^ neurons also via stimulating GHSR. In the presence of low (10 µM) concentration GHSR antagonist, the inward currents caused by sevoflurane could be reduced by 40% in the UCN1^+^/CART^+^ neurons (Figure S13h, Supporting Information). Interestingly, in the presence of a higher concentration (20 µM) of GHSR antagonist, the inward currents caused by sevoflurane could be completely blocked (**Figure** **5**a). We also explored the ionic mechanisms underlying the sevoflurane‐induced inward currents by conducting a slow ramp command test. The reversal potential of sevoflurane‐induced inward currents was ≈ −30 mV, which was very close to the equilibrium potential of the nonselective cation channels (NSCCs) (Figure 5b). GHSR activation stimulates the downstream phospholipase C‐protein kinase C pathway that has been reported to regulate NSCCs.^[^ ^44^ ^]^ Thus, we speculated that NSCCs might mediate the sevoflurane‐induced inward currents. Indeed, in the presence of NSCC blocker (FFA, 100 µM), the inward currents caused by sevoflurane could be reduced by 60% (Figure S13i, Supporting Information). When the concentration of NSCC blocker FFA was 300 µM, the sevoflurane‐elicited currents were nearly completely abolished (Figure 5c), indicating that the NSCCs were the major competent.

In addition, we used a GHSR structure‐based computational simulation to predict the GHSR binding sites for sevoflurane. GHSR belongs to Class A G‐protein‐coupled receptors, whose allosteric binding sites may be in contact with the orthosteric sites and located in the region of the TM domain.^[^ ^45^ ^]^ To predict the exact binding sites, we docked sevoflurane in different binding sites of GHSR, and found it could dock into the allosteric pockets under the orthosteric sites with binding free energy of −5.4 kcal mol^−1^. Its binding mode with orthosteric sites was also explored with a higher free energy of ‐4.9 kcal/mol, suggesting the allosteric pockets were more favorable for sevoflurane. In the sevoflurane/GHSR docking model, sevoflurane interacted with allosteric pockets by hydrophobic interactions with Thr276, Phe 272, Ala271, Cys275, Ser 315, Asn319, and Ile318 (Figure 5d).

We further tested whether other widely used general anesthetics, such as isoflurane, dexmedetomidine, midazolam, and propofol, can influence the activities of the UCN1^+^/CART^+^ neurons via GHSR. we first detected the c‐fos expression after anesthesia induced by isoflurane, dexmedetomidine, midazolam, propofol, or control condition with either oxygen or vehicle exposure alone. Dexmedetomidine, midazolam, and propofol increased the c‐fos expression level in the EWcp, but isoflurane had no such effect (Figure S14, Supporting Information). Additionally, we further carried out whole‐cell patch‐clamp recordings to observe the effects of these anesthetics on the activities of the sevoflurane‐activated neurons. For the neurons activated by sevoflurane, dexmedetomidine, midazolam, propofol, but not isoflurane, could exert excitatory effects (Figure S15,a–d, Supporting Information). The excitatory effects of propofol and midazolam were blocked by GHSR antagonist (Figure S15e,f, Supporting Information), while dexmedetomidine excited these neurons via enhancing the excitatory synaptic inputs (Figure S15b, Supporting Information), suggesting that GHSR in the UCN1^+^/CART^+^ cells is also the target for some of other general anesthetics including propofol and midazolam.

Last, whether GHSR in UCN1^+^/CART^+^ neurons is required for sevoflurane anesthesia was explored. It was locally infused GHSR antagonist (10 mM) into sevoflurane‐activated midbrain EW region. The EEG power spectrum to precisely judge whether the mice entered an anesthetic state was analyzed. Under this condition, GHSR antagonist increased the theta/delta power ratio during the induction of sevoflurane (2%) anesthesia, and delayed the time to anesthesia. Additionally, the time to recovery from sevoflurane anesthesia was also significantly shortened in the presence of the GHSR antagonist (Figure 5,e–h). These results indicate that GHSR activation is involved in sevoflurane anesthesia.

## 3 Discussion

In the study, we show that GHSR is highly enriched in sevoflurane‐activated midbrain UCN1^+^/CART^+^ cells. Ablation of the sevoflurane‐activated neurons or blockade of GHSR attenuates the anesthetic action of sevoflurane. These results provide novel cellular and molecular targets for sevoflurane anesthetisia.^[^ ^35^ ^]^

As reported in many previous studies,^[^ ^20^, ^21^, ^23^ ^]^ we found that EWcp contains multiple types of neurons including UCN1^+^/ CART^+^ neurons. Using traditional nonspecific manipulation, it has been reported that EWcp is related to addiction, attention, stress responses, and energy homeostasis.^[^ ^23^, ^24^, ^25^, ^26^, ^27^ ^]^ However, the behavioral role of different cell types in the EWcp is still unclear. Our results unveil a previously unappreciated role for EWcp UCN1^+^/CART^+^ neurons in sleep induction. These findings are supported by some clues reported in earlier studies. For example, long‐sleep mice have a greater density of UCN1 per cell and larger cells in the EWcp as compared to the short‐sleep mice. Additionally, activation of EWcp UCN1^+^/CART^+^ neurons has been reported to cause hypothermic effect that is also an important phenomenon in sleep.^[^ ^46^, ^47^ ^]^ Interestingly, a recent study showed that specific manipulation of EWcp CART^+^ cells enable maternal preparatory nesting without affecting sleep.^[^ ^26^ ^]^ One of the main reasons for this inconsistency may be that this recent study mainly used pregnant mice which showed a disrupted sleep‐wake cycle with decreasing total wake time and increasing NREM sleep time during late pregnancy.^[^ ^48^ ^]^

GHSR is expressed not only in the brain areas such as the pituitary, hypothalamus, and EWcp, but also in several peripheral tissues,^[^ ^49^, ^50^ ^]^ known to regulate not only growth hormone secretion but also food intake, glucose homeostasis, autonomic nervous system activity, and cognitive functions such as reward‐related behaviors.^[^ ^51^, ^52^ ^]^ Interestingly, many studies have reported that GHSR activation promotes sleep in several species, including rodents and humans after various routes of administration. For example, intravenous administration of growth hormone‐releasing peptide (GHRP)‐6 increased stage 2 of NREM sleep in humans.^[^ ^38^, ^53^ ^]^ Both oral activation of GHSR by MK‐677 or bolus injections of the ghrelin increased NREM sleep in young male subjects.^[^ ^37^, ^39^ ^]^ Additionally, systemic administration of ghrelin to mice also increased NREM sleep. This effect was absent in mice with nonfunctional GHSR.^[^ ^54^ ^]^ However, these findings are challenged by another study in rats.^[^ ^55^ ^]^ It has been reported that intracerebroventricular injection of ghrelin decreased NREM and REM sleep for 2 h. This decrease was followed by an enhancement in sleep during hours 3–12 after some doses of ghrelin. Based on these evidences, it is currently believed that a threshold in ghrelin concentrations exists for sleep. A slight increase during the night appears to promote sleep, while higher levels would disrupt sleep due to its stimulation of hunger. This idea was supported by several studies in humans, which showed that a low dose of 50 µg ghrelin injected around sleep onset increased sleep,^[^ ^37^ ^]^ while the nocturnal injection of 100 µg ghrelin increased hunger and food intake at night and disrupted sleep.^[^ ^56^ ^]^

Although there are links between sleep and ghrelin/GHSR signaling, the targets that mediate this effect remain unknown. Here we show that local injection of ghrelin into EW promotes NREM sleep, while local injection of the antagonist of GHSR has opposite effects, suggesting EW is an important node that mediates the sleep‐promoting effect of ghrelin/GHSR signaling. Interestingly, ghrelin levels in the blood retain a moderate increase during the night phase in humans.^[^ ^57^ ^]^ Additionally, sleep deprivation moderately elevated ghrelin levels.^[^ ^58^ ^]^ Therefore, it is reasonable to speculate that GHSR may sense this sleep‐related signal, and activate UCN1^+^/CART^+^ cells to promote sleep.

Over the past decades, it is widely considered that almost all general anesthetics including sevoflurane work by exerting a wide range of inhibitory impacts on cortical regions and subcortical wakefulness‐promoting nucleus.^[^ ^14^, ^59^, ^60^ ^]^ Recent studies reported the excitatory effects exerted by general anesthetics in sleep‐related neurons of the SON, and pain‐suppression circuit in the amygdala,^[^ ^13^, ^31^ ^]^ while the possible receptors that mediate these excitatory effects have not been identified. The present study shows that sevoflurane activates another sleep‐related EWcp nucleus through stimulating GHSR. Furthermore, midazolam and propofol, but not isoflurane or dexmedetomidine, also exert excitatory effects on the UCN1^+^/CART^+^ neurons via activating GHSR. These findings provide novel cellular and molecular mechanisms underlying general anesthesia and would be helpful for improved anesthesia, although further studies are needed to explore how these anesthetics activate GHSR through protein structure biology and the anesthetic effect after local knockout of *Ghsr* gene.

It is worth noting that the effect on anesthetic emergence is more prominent than that of the anesthetic induction which shows a great variation after inhibition of UCN1^+^/CART^+^ cells or blockade of EW GHSR. These results are consistent with previous studies, that is, often a single intervention in the wakefulness‐ or sleep‐related target of general anesthetics would only affect the recovery time, but not the induction time.^[^ ^8^, ^13^ ^]^ One possible explanation is that the activation of GHSR in EWcp region may be mainly involved in stabilizing anesthesia. This idea is supported by our in vivo electrophysiological data which showed that the discharge frequency of EW cells reached the highest mainly after entering anesthesia.

There are many other types of cells among and around the CART^+^/UCN1^+^ cells activated by sevoflurane, including glutamatergic, GABAergic, cholinergic, dopaminergic, and substance P neurons. Although sevoflurane activates the CART^+^/UCN1^+^ cells, the activities of some other cells were decreased during sevoflurane anesthesia. Future studies are needed to detect which types of cells are inhibited by sevoflurane and what effects of these cells exert on sevoflurane anesthesia. Additionally, it is still unclear what neurotransmitters CART^+^/UCN1^+^ neurons release. These neurons might not be glutamatergic, GABAergic, cholinergic, and monoaminergic neurons. Considering that these cells contain several neuropeptides, including CART, UCN1, and CCK, it is very likely that these neurons might be peptidergic. While future studies are needed to identify the specific type of neuropeptides released by these cells.

Sleep is a reversible physiological process, the occurrence of which is due to biological rhythms and sleep homeostasis factors activate the sleep‐promoting nuclei and then inhibit the wakefulness‐promoting nuclei.^[^ ^29^, ^61^ ^]^ General anesthesia is the action of exogenous drugs on the brain to make it enter the unconscious state.^[^ ^14^ ^]^ Although many differences exist between sleep and general anesthesia, a large number of literature have reported that anesthetics work by affecting the sleep/wakefulness‐promoting nuclei. On one hand, anesthetics work through exerting an inhibitory impact on wakefulness‐promoting nuclei, such as the ventral tegmental area, orexinergic neurons in the hypothalamus, medial parabrachial neurons, and nucleus accumbens.^[^ ^7^, ^12^, ^14^, ^16^, ^62^ ^]^ On the other hand, anesthetics can activate the sleep‐promoting nuclei.^[^ ^13^ ^]^ Through these dual mechanisms, anesthetics can inhibit the brain functional networks and make it into an unconscious state.

Although the nuclei that control sleep/wakefulness are the targets of the anesthetics, sleep, and action of anesthetics are different. These differences may be mainly due to the way and extent of affecting the wakefulness/sleep‐promoting nuclei. Under physiological condition, a moderate increase in sleep homeostasis factors such as adenosine activates sleep‐promoting nuclei. After entering sleep, the concentration of adenosine would rapidly decrease. The dynamic changes of sleep homeostasis factors lead to the reversibility of sleep.^[^ ^63^, ^64^ ^]^ However, exogenous anesthetics not only continuously act on sleep‐promoting nuclei, but also directly inhibit wakefulness‐promoting nuclei and neocortical regions, such as frontal and parietal cortices, by potentiating GABA_A_Rs.^[^ ^6^, ^7^, ^12^, ^13^, ^14^ ^]^ Sevoflurane could inhibit N‐methyl‐D‐aspartate ionotropic glutamate receptors and affect the potassium channel activity.^[^ ^65^ ^]^ Through these mechanisms, they might exert a stronger and more persistent inhibition of brain functional network compared with the sleep state and make the subjects in anesthesia unable to be awakened.^[^ ^12^, ^14^ ^]^ Here we identified the sleep‐related GHSR signaling in EWcp UCN1^+^/CART^+^ cells involved in the sevoflurane anesthesia. These findings further suggest that the multiple subcortical nuclei and molecular targets that control brain states are involved in the anesthetic action. Although GHSR is highly expressed in the EWcp, it should be noted that GHSR is also moderately expressed in other sleep‐wake regulating nuclei like the ventral tegmental area (VTA) and parabrachial nucleus (PB).^[^ ^36^, ^66^ ^]^ While we found that sevoflurane did not significantly affect the number of c‐fos^+^ neurons in these brain regions. One explanation is that the weak expression of GHSR might result in that sevoflurane cannot remarkably affect the activity of cells in these brain areas. Anyhow, further studies are needed to assess whether GHSR activation in other sleep‐wake regulating nuclei contributes to sevoflurane anesthesia.

Clinical researchers have shown that sevoflurane has unique advantages in many investigations. For instance, induction of anesthesia with sevoflurane is more favorable in pediatric patients in comparison with other anesthetics due to its lack of airway irritation.^[^ ^67^, ^68^ ^]^ It has great potential to maintain hemodynamic stability with a significantly low incidence of cardiac events, and has been reported to have a favorable recovery profile.^[^ ^3^, ^69^, ^70^, ^71^ ^]^ These studies lead to the idea that sevoflurane may be an ideal anesthetic agent among various anesthetics. We found that midbrain sleep‐related UCN1^+^/CART^+^ cells are involved in the sevoflurane anesthesia. This functional property may allow sevoflurane anesthesia to possess some characteristics close to physiological sleep, granting it unique advantages such as reduced side effects and enhanced recovery profiles.

## 4 Conclusion

In conclusion, here we show that sevoflurane activated the UCN1^+^/CART^+^ neurons in the midbrain to induce and maintain the state of anesthesia. GHSR is enriched in the sevoflurane‐activated UCN1^+^/CART^+^ cells. Ablation or inhibition of the sevoflurane‐activated neurons, or blockade of GHSR, significantly attenuates the anesthetic effect of sevoflurane. Although further studies are needed to explore how sevoflurane activates GHSR through protein structure biology, these findings provide a novel target including the nucleus and receptor in the field of sevoflurane anesthesia.

## 5 Experimental Section

### Animals

All animal care and experimental procedures were approved by the Army Medical University Guide for the Care and Use of Laboratory Animals. Adult C57BL/6J male mice were provided by the Laboratory Animal Center at the Army Medical University. Transgenic Fos^2A‐iCreERT2^ mice were purchased from the Jackson Laboratory (JAX: 03 0323). C57BL/6J mice aged 4–8 weeks old were used for brain slice preparation. For behavioral tests and morphological experiments, adult C57BL/6J mice aged 8–12 weeks old were used. All experimental animals were housed in a 12 h light/12 h dark cycle with lights on at 8:00 A.M., except for the mice that were used for polysomnographic recordings with lights on at 6:00 A.M. The room temperature was kept constant with food and water ad libitum.

### Stereotaxic Surgery

Mice was anesthetized with isoflurane and placed in a stereotaxic apparatus (RWD Life Technology Co, Ltd., China). Ophthalmic ointment was used to prevent the eyes from drying. It was carried out standard surgery to expose the brain surface above the EW. For implantation of electrode array, coordinates were as follows: anteroposterior (AP) ‐3.60 mm, mediolateral (ML) 0.00 mm, and dorsoventral (DV) −3.00 mm. For cannula implantation, coordinates were as follows: AP ‐3.55 mm, ML 1.10 mm, and DV −3.20 mm at an angle of 20° toward the midline. For EEG and EMG recordings, two screws into the skull on top of the left and right cortex was inserted, at AP −3.50 mm, ML 3.00 mm and AP 1.50 mm, ML 1.50 mm, respectively.^[^ ^35^ ^]^ A reference screw was inserted into the skull on top of the cerebellum. Also, two EMG electrodes were placed into the dorsal neck musculature. Dental acrylic was used to fix the cannula and electrodes to the skull. The same coordinates for virus injection, as for the implantation of electrode array were used. Nanoject II (Drummond Scientific, Broomall, PA) was used to inject virus with a micropipette opening ≈20 µm. 3 min after pipette insertion, ≈80 nl virus was injected gradually in 3 min. 3 min after injection, the pipette was withdrawn slowly.

### Immunofluorescence

Immediately after being anesthetized with isoflurane, mice were transcardially perfused with 0.1 M PBS, followed by ice‐cold 4% paraformaldehyde (PFA) in PBS. Brains were carefully extracted from the skull and further postfixed in 4% PFA at 4 °C for 2 h, then transferred into 30% sucrose in PBS for dehydration at 4 °C overnight. Brains were then continuously sectioned into 40 µm coronal slices using a freezing microtome (CM 3050S, Leica). Sections were washed twice with PBS, followed by incubation in blocking solution (Beyotime, China) with or without 1% Triton X‐100 at 37 °C for 30 min. Then the sections were incubated in appropriate primary antibodies in blocking solution at 4 °C overnight. Primary antibodies were applied as follows: rabbit anti‐c‐fos (1:1000, ab190289, Abcam), rat anti c‐fos (1:1000, 226 017, Synaptic Systems), mouse anti‐ChAT (1:200, AMAb 91 130, Sigma‐Aldrich), rabbit anti‐Tph2 (1:200, AMAb 91 108, Sigma‐Aldrich), rabbit anti‐TH (1:200, AMAb 91 108, Sigma‐Aldrich), mouse anti‐NET (1:200, AMAb 91 116, Sigma‐Aldrich), rabbit anti‐ HDC (1:500; Progen, Germany), rabbit anti‐NeuN (1:500, 24 307, Cell Signaling Technology). Afterward, sections were washed 3 times with PBS, then transferred into secondary antibodies in PBS and incubated at room temperature for 2 h. Secondary antibodies were used as follows: Alexa Flour 488 donkey anti‐rabbit IgG (1:500, Invitrogen), Alexa Flour 594 donkey anti‐rat IgG (1:500, Invitrogen), Alexa Flour 568 donkey anti‐rabbit IgG (1:500, Invitrogen) and Alexa Flour 488 donkey anti‐mouse IgG (1:500, Invitrogen), Alexa Flour 647 donkey anti‐rabbit IgG (1:500, Invitrogen). After washing 3 times with PBS, sections were finally mounted and coverslipped. Fluorescence images were taken using a microscope (Olympus), digital slide scanner (SlideView VS 200, Olympus), or confocal microscope (LSM 780 or LSM 880, Zeiss).

### Fluorescence In Situ Hybridization (FISH)

Mice were anesthetized and transcardially perfused with 0.1 M PBS followed by 4% PFA in PBS. Brains were then placed in 4% PFA at 4 °C for 24 h for postfixation, followed by gradient dehydration in sucrose (10%, 20%, and 30%, respectively). After freezing at −20 °C at least for 1 h in the freezing microtome, brains were sectioned into 14 µm coronal slices. FISH was performed using RNAscope Multiplex Fluorescent Assays V2 (Advanced Cell Diagnostics (ACD)) according to the manufacturer's instructions, including the following probes: *Slc17a6* (319 171, ACD), *Slc17a7*(416 631, ACD), *Slc17a8*(431 261, ACD), *Calca* (417 961, ACD), *Gad1* (400 951, ACD), *Gad2* (439 371, ACD), *Cck* (402 271, ACD), *Fos* (316 921, ACD), *Cartpt* (432 001, ACD), *Ucn1* (466 261, ACD) and *Tac1*(410 351, ACD). Fluorescence images were taken using a confocal microscope (LSM 780 or LSM 880, Zeiss).

### Whole‐Cell Patch Clamp Recording

Male C57 BL/6J mice aged 4–8 weeks were used for brain slice preparation by methods that have been described in detail in the previous study.^[^ ^72^ ^]^ Briefly, coronal brain slices (300 µm) containing the EW were prepared with a vibroslicer (VT1000, Leica Microsystems) in an ice‐cold artificial cerebrospinal fluid (ACSF) (composition in mM: 119 NaCl, 2.5 KCl, 1.2 NaHPO_4_, 25 NaHCO_3_, 125 glucose, 2 MgCl_2_, 2 CaCl_2_) continuously superfused with 95% O_2_ and 5% CO_2_. Slices were incubated for 15 min at 32°C in an incubation fluid (composition in mM: 110 NMDG, 2.5 KCl, 1.2 NaH_2_PO_4_, 25 NaHCO_3_, 25 glucose, 10 MgSO_4_, 0.5 CaCl_2_, adjusted to pH 7.3 with HCl) with oxygenation (95% O_2_–5% CO_2_). Slices were then transferred to ACSF and continuously aerated with 95% O_2_ 5% CO_2_ for at least 40 min at room temperature (20—24 °C) before recording. During recording, slices were submerged in oxygenated ACSF in a recording chamber.

Targeted neurons were identified with an upright microscope with Leica differential interference contrast optics and an infrared video imaging camera. Patch electrodes pulled to tip resistances of 3–5 MΩ using a multistage puller (Sutter Instruments) were used in whole cell recordings and were filled with an internal solution (composition in mM: 125 potassium gluconate, 20 KCl, 10 HEPES, 1 EGTA, 2 MgCl_2_, 4 ATP, adjusted to pH 7.2–7.4 with KOH). Neurons were held at a membrane potential of −60 mV and were excluded from analysis if the series resistance increased by >15% during recording or exceeded 25 MΩ. After at least 5 min of stabilization, data were obtained with an EPC 10 amplifier (HEKA Elektronik, Lambrecht/Pfalz, Germany) and analyzed with Pulse/Pulsefit v.8.74 (HEKA Elektronik) and Igor Pro v.4.03 (WaveMatrics). To investigate the effect of sevoflurane, sevoflurane was puffed on the slice for 2 min. Action potentials were determined automatically by using Mini‐analysis software (version 6.0, Synaptosoft).

### Drugs for Whole‐Cell Patch Clamp Recording

6‐cyano‐7‐nitroquinoxaline‐2, 3‐dione (CNQX), D‐2‐amino‐5‐phosphonovaleric acid (APV) and picrotoxin (PTX) were obtained from Sigma (St Louis, MO). Human ghrelin peptide was purchased from Abcam (ab73131). [D‐Lys^3^]‐GHRP‐6 was purchased from Tocris (136054‐22‐3). CNQX and PTX were dissolved in dimethyl sulfoxide, other drugs were dissolved in ACSF. All drugs were prepared as concentrated stock solutions and either added immediately to the recording ACSF or internal solution at working concentrations (1:1000) and frozen at −20 °C until use. Dimethyl sulfoxide, diluted 1:1000 in ACSF, had no effect on either holding current or synaptic currents.

Sevoflurane was sonicated into solution in preoxygenated ACSF (50 ml) to make 0.03%, 0.08%, and 0.4% (*v/v*) sevoflurane. 0.4% sevoflurane (*v/v*) was selected to further investigate its molecular mechanisms. The selection of these concentrations of sevoflurane was in line with previous studies and clinically relevant.^[^ ^13^, ^40^, ^41^ ^]^ To test the effect of ghrelin, 10–500 nM of ghrelin concentration was selected. The sevoflurane and ghrelin were bathed in the acute brain slice for 2 min to test their effects on neuronal activities.

### Single Cell Reverse Transcription PCR (scRT‐PCR)

After whole cell recording, cytoplasm was sucked into an electrode tube containing 7 ul of internal solution and then expelled into the 200 ul PCR tube (Axygen, USA) as described previously. The cDNA of each cell was made using the PrimeScript II 1st Strand cDNA Synthesis Kit (6210A, TaKaRa) following the manufacturer's instructions. In brief, after denaturing at 65 °C for 5 min, the reaction mixture containing cytoplasm of each cell was cooled on ice and then added to the reverse transcription system containing the Oligo dT primer and random primers. The reaction was performed as follows: 60 min at 45 °C and 15 min at 70 °C. The resulting cDNA was stored at −20 °C or used for detecting the presence of mRNAs coding for *β*‐actin, UCN1, and VGLUT2 in the same cell. The first round of PCR amplification was performed with the gene‐specific multiplex primers using the SuperScript III One‐Step RT‐PCR Kit (12 574 026, Invitrogen). cDNA (1–2 ul) synthesized in the reverse transcription step was used as the template for the first round of PCR amplification, the reaction was carried out as follows: 2 min at 94 °C; 40 cycles of 15 s at 95 °C, 30 s at 55 °C, and 30 s at 68 °C; 5 min at 68 °C. Next, PCR was performed with nested primers for each gene and the first PCR product (1–2 ul) was used as the template. The second reaction was carried out as follows: 2 min at 94 °C; 40 cycles of 15 s at 95 °C, 30 s at 55 °C, and 30 s at 68 °C; 5 min at 68 °C. Finally, the amplification products were visualized by electrophoresis in 2% agarose gels.

Primers (5’‐3’) and product sizes for single‐cell RT‐PCR:
*β‐actin* (sense/anti‐sense):
multiplex, 5’ gacccagatcatgtttgagacc/ gctaggagccagagcagtaatct;
nested, aggctgtgctgtccctgtatg/ gaggtctttacggatgtcaacg
Final product 470 bp
*Ucn1* (sense/anti‐sense):
multiplex, 5’ tggtggcgttgctgctctt/tatgatgcggttctgctctgc;
nested, tgctcttggcacagcttcg/aaggtgaggtcgatggacagtg
Final product 232 bp
*Slc17a6 (*encoding VGLUT2) (sense/anti‐sense):
multiplex, 5’ tgttctggcttctggtgtcttacgagag/ttcccgacagcgtgccaaca;
nested, tcaacaacagcaccatccac/gggctctcgtaagacaccag
Final product 315 bp
*Ghsr* (sense/anti‐sense):
multiplex, 5’ accaccaacctctacctatccag/agcacagtgaggcagaagacc
nested, gctacttcgccatctgcttc/cgacacccataccatcacg
Final product 230 bp

### Fiber Photometry

Fluorescence signals of GcaMP6s were recorded with a fiber photometry system (Inper Technology Co., Ltd) as described in previous studies.^[^ ^28^ ^]^ Briefly, after AAV2/9‐Ef1a‐DIO‐jGCaMP6s injection in EW region and the Fos‐TRAP procedure, an optical fiber (diameter: 200 µm, NA: 0.48, Doric lenses, Quebec, Canada) was carefully placed above the EW (coordinates, bregma: AP = −3.60 mm; ML = 0.0 mm; DV = −2.90 mm) in the adult Fos^2A‐iCreERT2^ mice, connected to 410 and 488 nm laser. The calcium activity‐dependent GCaMP6s fluorescence signal (488 nm) and calcium activity‐independent fluorescence (410 nm) were simultaneously captured by a CMOS camera, providing an internal control for movement and bleaching artifacts. The laser power at the tip of the optical fiber was set to 10–20 µW to minimize bleaching. To examine the alterations of neuronal activity in sevoflurane anesthesia, synchronized recordings of calcium signals, EEG and EMG were obtained before, during, and after sevoflurane exposure (2%, 15 min). Values of fluorescence changes were converted to Δ*F*/*F* by calculating (*F* − *F*0)/*F*0, where Δ*F* is the variation of fluorescence between each sampling and *F*0 is the averaged baseline fluorescence.

### Multiple‐Channel Single Unit Recording

Multiple‐channel single‐unit recording in the midbrain EW region was in accordance with previous studies.^[^ ^28^, ^73^ ^]^ Adult C57BL/6J male mice (*n* = 16) were used for multiple‐channel single‐unit recording. The mice were housed with food and water available ad libitum before surgery. All the experiments were conducted in the light phase (12:00–18:00).

For multiple‐channel single‐unit recording of midbrain EW neuronal activity during anesthesia and the spontaneous sleep‐wake cycle, a custom‐made electrode array was aimed into the midbrain EW region. The electrode array used in the present study contained two tetrodes. Each tetrode was constructed using 4 nickel‐cadmium wires (17 µm). Implantation of the electrode was described in previous study in detail.^[^ ^28^, ^73^ ^]^ Briefly, the electrode was slowly advanced into the targeted region by using a piezoelectric micromanipulator (Sensapex). Single‐units were monitored during the surgery in real time to obtain the fine signal‐to‐noise ratio of spike activities. To monitor EEG‐EMG activity combined with single‐unit recording, one small craniotomy hole was drilled at the frontal region (coordinates, bregma: AP = +1.50 mm; ML = +1.50 mm) and one stainless miniature screw was carefully inserted into the hole above the frontal cortex. Two EMG electrodes were placed between the neck musculature. The reference electrode was a small stainless screw, which is fixed in the skull above the cerebellum. All the electrodes including tetrodes, EEG, EMG, and reference electrodes were previously soldered to a micro pin connector. Dental cement was used to tightly secure the electrodes to the skull.

After electrode implantation, mice were allowed to recover for at least one week, and electrodes were connected to a 16‐channel headstage with operational amplifiers to minimize cable movement artifacts. Signals were acquired with an amplifier (Digital Lynx SX; Neuralynx, MO, USA) and Cheetah acquisition software (Cheetah 5.7.4), and digitized at 30 kHz, and band‐pass filtered (0.1–9 kHz).

### Firing Frequency Analysis

Single‐unit spike sorting using offline sorter software (version 3.0), as described in previous published work was conducted.^[^ ^28^, ^73^ ^]^ First, principal component analysis was conducted to extract and represent the first 2 principal components on a 2‐dimensional plot of detected spike events. Waveforms with similar principal components were clustered using *k*‐means clustering algorithm. The isolated cluster as a single unit recorded from the same neuron was considered. Cross‐correlation histograms was used to eliminate cross‐channel artifacts and discarded units with interspike intervals less than 2 ms. The firing rate histograms before, during, and after sevoflurane application were produced using NeuroExplorer software (version 5.0).

After recording the single units in EW during sevoflurane anesthesia, the changes of discharge activities during wakefulness and sleep in some animals (*n* = 10) were further detected. In these animals, synchronous EEG‐EMG and EW single‐units during the light phase, when mice spend the most time in sleep were recorded. NeuroExplorer software (version 5.0) was used to score behavioral states across consecutive nonoverlapping 4 s epochs. Wakefulness as desynchronized, low‐amplitude EEG rhythms and elevated EMG activity; NREM sleep as synchronized, high‐amplitude and low frequency (0.5–4 Hz, delta) EEG activity and lower EMG activity when compared with wakefulness. REM sleep was defined as containing a pronounced theta (4–10 Hz) rhythm with nearly no EMG activity.

Criteria for identification of the same single units during sevoflurane anesthesia, and wakefulness and sleep: 1) The unit has the similar cluster boundaries in a 2‐dimensional plot of the first 2 principal components, and 2) the waveforms obtained on all four wires of one tetrode must be identical. Peri‐state transition (wake to NREM, NREM to REM) line plots of neuronal firing rates were calculated with a bin size of 1 S. After collection of electrophysiological data, the mice were deeply anesthetized with isoflurane and underwent electrolytic lesion (0.05 mA). Brains were kept for histological analysis, and only included for data analysis in the animals where the inserted electrode was restricted to the EW region.

### EEG‐EMG Recordings and Sleep‐Wake State Classifications

For pharmacological activation or blockade of GHSR signal, adult mice were anesthetized with isoflurane and then implanted with EEG‐EMG electrodes and cannula. All electrodes were welded to a micro‐pin connector. The EEG‐EMG electrodes and cannula were affixed to the skull with dental cement. After recovery from anesthesia, mice were individually placed in a sleep recording box equipped with a signal receiver board and an overhead camera, and then underwent polysomnographic recordings. The EEG‐EMG signals were amplified (Grass Link, Grass Technologies) and band‐pass filtered (EEG: 0.3‐30 Hz, EMG: 10–100 Hz). Then the signals were digitized at 128 Hz using sleep recording software (Vital Recorder, Kissei Comtec).

The polygraphic recording signal was automatically analyzed based on spectral signatures of EEG‐EMG waveforms with the sleep analysis software (SleepSign for animals, Kissei Comtec). States were classified across consecutive nonoverlapping 4s epochs. Wakefulness was defined as desynchronized, low‐amplitude EEG rhythms and elevated EMG activity with phasic bursts. NREM sleep was defined as synchronized, with high amplitude and low frequency (0.5–4 Hz, delta) EEG activity and lower EMG activity compared with wakefulness with no phasic bursts. REM sleep was defined as containing a pronounced theta (4–10 Hz) rhythm with nearly no EMG activity. The scoring results were inspected visually and corrected manually, if necessary. Sleep‐wake state classifications were performed by an experienced investigator who was blind to the experimental manipulations.

For pharmacological administration, ghrelin peptide was dissolved in ACSF, and the final concentrations were 10 nM and 100 nM. Ghrelin peptide or ACSF were administered during the light phase (18:00, ZT12). GHSR antagonist [D‐Lys^3^]‐GHRP‐6 (Tocris, 136054‐22‐3) was dissolved in ACSF, the final concentrations were 2 mM and 10 mM. [D‐Lys^3^]‐GHRP‐6 or ACSF was administered at (6:00, ZT0). After injection (total volume 200 nl at a rate of 100 nL min^−1^ for 2 min), the needle was left for another 2 min to allow for full infusion. EEG‐EMG recordings were started immediately after drug administration and continued for 3 h. Each mouse was administered with ghrelin peptide/[D‐Lys^3^]‐GHRP‐6 or ACSF separated by at least a 3‐day washout period. Experimenters were blind to the drugs administered by using a cross‐over design.

After the collection of behavioral data, the mice were deeply anesthetized, and brains were removed and underwent DAPI staining for histological analysis. Only animals in which the inserted cannula was restricted in the EW were included for data analysis.

### Fos TRAP Procedure

One week after virus injection, including either AAV2/9‐hSyn‐DIO‐mCherry (Brain Case, China) or AAV2/9‐hSyn‐DIO‐hM4D(Gi)‐mCherry (Brain Case, China) or AAV2/9‐Ef1a‐DIO‐DTA (Brain Case, China) plus AAV2/9‐hSyn‐DIO‐mCherry, the adult Fos^2A‐iCreERT2^ mice were intraperitoneally injected with tamoxifen (T‐5648, Sigma‐Aldrich) in corn oil (20 mg ml^−1^) at a dose of 150 mg per kg for 5 consecutive days.^[^ ^74^, ^75^ ^]^ The mice was then exposed to 2% sevoflurane in oxygen for 2 h to label the sevoflurane‐activated neurons. For chemogenetic inhibition and ablation, EEG and EMG electrodes were implanted into the skull of these mice, and exposed the animals to a general anesthesia assay one week later. For TRAP efficiency assay, mice were re‐exposed to 2% sevoflurane in oxygen for 2 h one week after tamoxifen injection, followed by perfusion and c‐fos staining.

### General Anesthesia Assay

All the general anesthesia experiments were performed between circadian nomenclature ZT6‐ZT12. With simultaneous video plus EEG and EMG recording, mice were placed in a gas‐tight recording chamber connected to a standard sevoflurane vaporizer on one side and a waste gas absorption system (RWD Life Technology Co, Ltd., China) on the other side. Similar to those previous studies,^[^ ^7^, ^13^ ^]^ 2% sevoflurane in oxygen was continuously delivered into the chamber for 20 min after an initial 5 min of baseline recording (continuously infused with a flow of 100% oxygen (1.5 L min^−1^)). Mice were then allowed to emerge from GA for several minutes (still continuously infused with 1.5 L min^−1^ 100% oxygen to avoid hypoxia).

For chemogenetic inhibition, mice randomly was pretreated with saline (i.p.) or CNO (3 mg kg^−1^, i.p.) 30 min before sevoflurane anesthesia. To specifically modulate GHSR activity, either GHSR antagonist [D‐Lys^3^]‐GHRP‐6 (10 mM, 500 nl) or agonist ghrelin (100 nM, 500 nl) was locally delivered into EW through an intracranially‐inserted cannula and infused at a rate of 150 nl min^−1^ by a syringe pump (Harvard apparatus) in C57 BL/6J mice 20 min before sevoflurane anesthesia. vehicle was administered as a control. After infusion, the needle for another 2 min was left to allow for full infusion.^[^ ^76^ ^]^ Each drug was given to each mouse with at least 3 days of washout between each experiment. Experimenters were blind to the drugs administered by a cross‐over design. Only animals in which the region of viral transduction or the inserted cannula was restricted in the EW were included for data analysis.

### Calculating the time to Deep Anesthesia

Whether the animals entered the state of deep anesthesia by precisely analyzing the EEG power spectrum was judged. EEG delta (0.5–4 Hz) power gradually increases from awakening to anesthesia, while the EEG theta (4‐12 Hz) power decreases from awakening to anesthesia. The burst suppression represents a very downregulation of cortical activity, where 95% of cortical cells are silent, and therefore mice are deeply unconscious during burst suppression.^[^ ^7^, ^32^, ^33^ ^]^ When reaching deep anesthesia, burst suppression continuously occurred. The ratio of EEG theta wave to delta wave power (theta/delta ratio) was calculated, and a simple exponential function was used to fit to the theta/delta ratio y against the time x as

(1) $$y=a\cdot e^{b\cdot x}$$

where a and b are two fitting parameters, and parameter b is defined as the anesthesia speed. According to these fitting parameters, the time point when the theta/delta ratio decreased to 0.3 was taken, which is the average theta/delta ratio of the burst suppression, as the time point of entering deep anesthesia.

### Loss and Recovery of Righting Reflex

Loss and return of righting reflex were assessed as previously described.^[^ ^7^, ^77^ ^]^ Briefly, after virus injection in EW region and the Fos‐TRAP procedure, the adult Fos^2A‐iCreERT2^ mouse was placed into the anesthesia chamber and exposed to 2% sevoflurane in oxygen (2 L min^−1^) for 20 min. To assess LORR, the chamber was rotated 180°every 30 s after sevoflurane delivery until the mouse was unable to turn itself prone onto all four feet. All the mice were kept under observation until the return of the righting reflex after sevoflurane discontinuation, determined by all four paws touching the flour.

### GHSR Structure‐Based Computational Simulation to Predict Docking Sites

The docking study was performed using Autodock. In this calculation, the free open‐source package Autodock41 (AD4) and Autodock Vina2 (Vina) were employed to calculate the ligand‐binding affinity. The X‐ray structure of Ghrelin receptor binding with an agonist was downloaded from RSCB PDB (https://www.rcsb.org/structure/7F9Z). The orthosteric docking site was set as center_*x* = 112.483, center_*y* = 109.302, center_*z* = 119.334 with a grid size of 25 Å × 25 Å × 25 Å, which is a large cavity enough to cover the entire target active site. The allosteric docking site was set as center_*x* = 103.483, center_*y* = 93.902, center_*z* = 104.334 and the grid size was set to 15 Å × 15 Å × 15 Å, which is a large cavity enough to cover the entire target active site. The largest ligand‐binding affinity was determined to have the best docking results.

### General Anesthetics Administration for c‐Fos Survey

All the anesthetics were used according to previous studies.^[^ ^13^, ^78^ ^]^ Dexmedetomidine (Sigma‐Aldrich, SML0956) was dissolved in PBS and injected intraperitoneally (100 µg kg^−1^). Propofol (Sigma‐Aldrich, D126608) was dissolved in PBS and injected intraperitoneally (180 mg kg^−1^). Midazolam (Nhwa, China, MZ220614) was dissolved in PBS and intraperitoneally injected (50 mg kg^−1^). Isoflurane (1.2%, RWD Life Science, R510‐22) or sevoflurane (2%, AbbVie, NDC 66794‐015‐25) was continuously delivered in oxygen (1.5 L min^−1^). Either the vehicle intraperitoneal injection or oxygen exposure alone was performed as a control.

### Data Analysis and Statistics

All data are presented as mean ± SEM. Normality and equal variances were formally tested for each group of data. For data with normal distribution and equal variances, paired or unpaired *t*‐tests, and analysis of variance (ANOVA) with post hoc test were performed for the comparisons between groups. If data did not conform to the normal distribution, Nonparametric tests were performed. Significant differences were accepted when *P* < 0.05.

## Conflict of Interest

The authors declare no conflict of interest.

## Author Contributions

T.T.Y., N.W., and J.H. contributed equally to this work. Z.A.H., H.L., and C.H. conceived the study. Z.A.H., H.L., C.H., X.J.X., and S.C.R. designed the experiments. T.T.Y., N.W., J.H., Y.L.W., Y.X.L., Y.W.H., X.L., Y.Q.O., F.L.L., Z.Y.Z., Y.L., R.R., X.Q., and C.H. executed the experiments and conducted statistical analysis. T.T.Y., N.W., Z.A.H., and C.H. wrote the paper with the help of X.D.T., Y.J.W., X.W.C., Z.X.Y.. All authors read and commented on the manuscript.

## Supporting information

Supporting Information

## Data Availability

The data that support the findings of this study are available from the corresponding author upon reasonable request.
