# Supplementary material for: A Sleep‐Specific Midbrain Target for Sevoflurane Anesthesia
Source: Adv Sci (Weinh). 2023 Mar 24;10(15):2300189. doi: 10.1002/advs.202300189 (PMC10214273; doi:10.1002/advs.202300189)
Supplement: Supplementary file 1 — Supporting Information [file ADVS-10-2300189-s001.pdf]

Supporting Information

Title A Sleep-Specific Midbrain Target for Sevoflurane Anesthesia

Tingting Yi†, Na Wang†, Jing Huang†, Yaling Wang, Shuancheng Ren, Jianxia Xia, Yiwen Hu, Yixiang Liao, Xin Li, Fenlan Luo, Qin Ouyang, Yu Li, Ziyi Zheng, Qin Xiao, Rong Ren, Zhongxiang Yao, Xiangdong Tang, Yanjiang Wang, Xiaowei Chen, Chao He, Hong Li*, Zhian Hu**

†These authors contributed equally.

*Corresponding authors: zhianhu@aliyun.com (Zhian Hu, lead contact); lh78553@163.com (Hong Li); hechaochongqing@163.com (Chao He).

This file includes:

Figures S1 to S15.

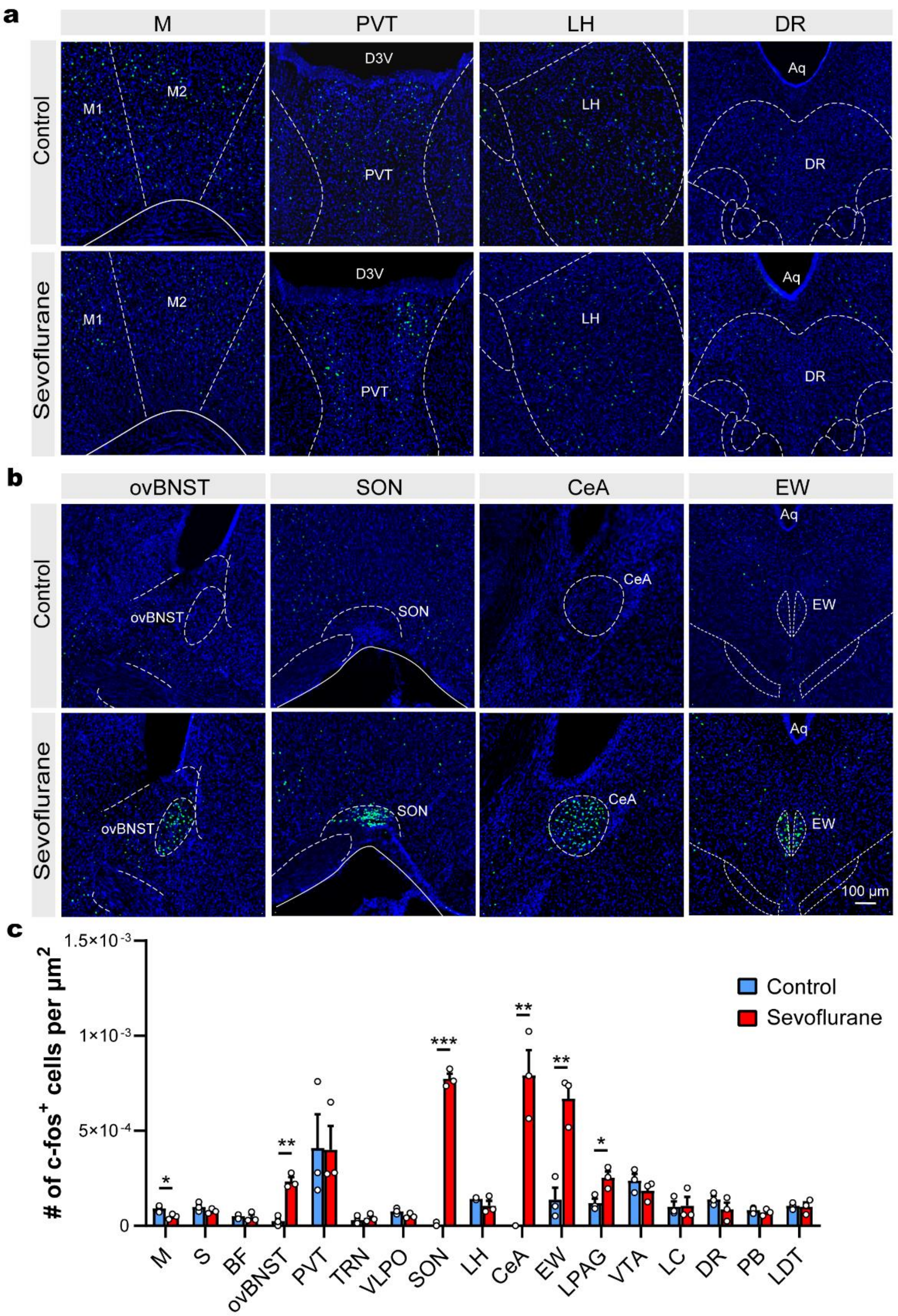

Figure S1. An unbiased brain-wide map of c-fos expression after a period of sevoflurane anesthesia. a,b) Representative immunofluorescence (IF) images showing the brain regions without and with an increase of c-fos expression levels after a period of sevoflurane anesthesia (2 h, 2%). c) Histograms summarized the c-fos expression levels in the cortical and subcortical areas after sevoflurane anesthesia (mean ± SEM). Unpaired t test, **P* < 0.05, ***P* < 0.01, ****P* < 0.001, n = 3 mice. M, primary motor cortex. S, primary sensory cortex. BF, basal forebrain. ovBNST, oval division of the bed nucleus of the stria terminalis. PVT, paraventricular nucleus of thalamus. TRN, reticular nucleus of thalamus. VLPO, ventral lateral preoptic area. SON, supraoptic nucleus. LH,

lateral hypothalamus. CeA, central amygdala. EW, Edinger-Westphal nucleus. LPAG, lateral periaqueductal gray. VTA, ventral tegmental area. LC, locus coeruleus. DR, dorsal raphe. PB, parabrachial nucleus. LDT, laterodorsal tegmental nucleus.

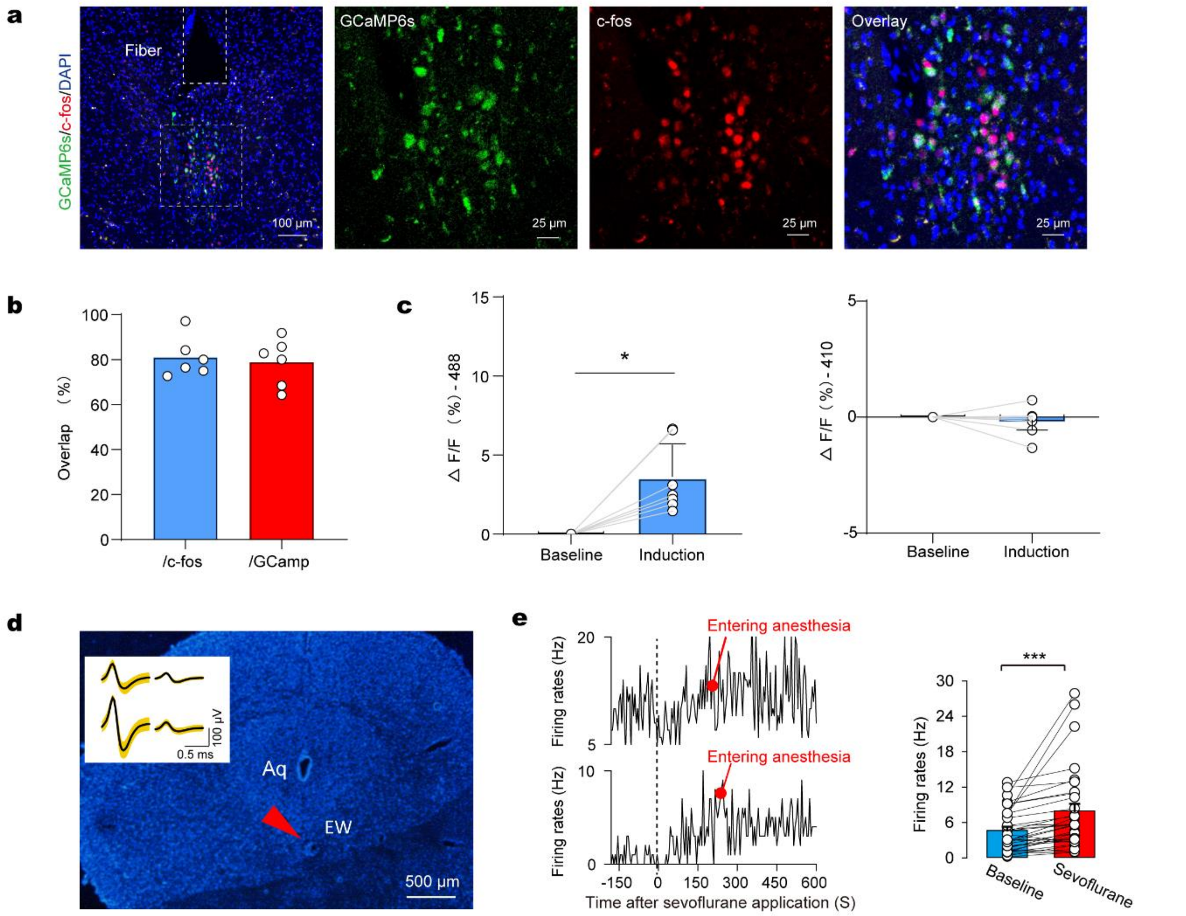

Figure S2. Assessing the activity of the sevoflurane-activated neurons during anesthesia by photometry and multi-channel single-unit recording. a) GcaMP6s/c-fos immunofluorescence in EW neurons and track of the optic fiber implanted above EW. b) Histograms showing the percentage of overlap in c-fos-positive and GcaMP6s-positive neurons, respectively. Data for each group were obtained from 3 mice. c) Further analysis of quantification of Ca^{2+} signal changes during baseline and induction based on the critia of burst supression onset (mean ± SEM). Wilcoxon matched-pairs signed rank test, * *P* < 0.05, n = 7 mice. d) 4,6-diamidino-2-phenylindole, dihydrochloride (DAPI) staining showing the electrode planted in the sevoflurane-activated midbrain region. Red arrow indicates the recording position. Aq, aqueduct of midbrain. Inset, the image showing the sorted waveforms from a recorded EW neuron. d) Left, The firing rates of two examples of sevoflurane-activated neurons before and after application of sevoflurane. Right, average firing rates of sevoflurane-activated neurons during the baseline and after entering anesthesia state (mean ± SEM). Wilcoxon signed-rank test, *** *P* < 0.001, n = 33 neurons.

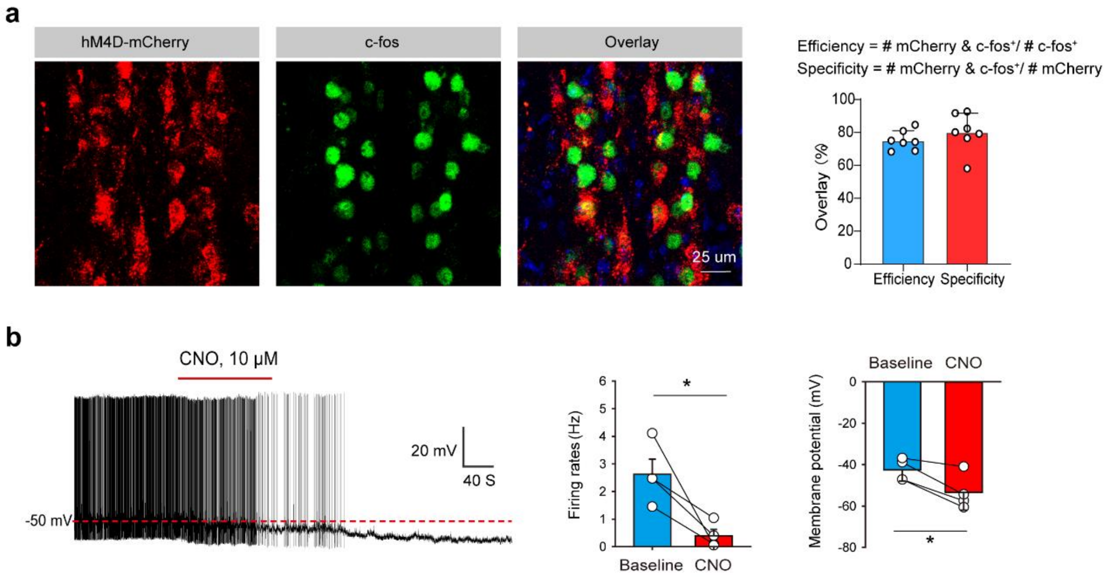

Figure S3. Targeted recombination in active populations (TRAP) technology to specifically inhibit the sevoflurane-activated neurons. a) Left, representative IF images showing the expression of c-fos and mCherry in the midbrain EW region. Right, histograms showing the specificity and efficiency of TRAP technology. Data for each group were obtained from 3 mice. b) Left, CNO caused membrane hyperpolarization in sevoflurane-activated neurons. Middle, CNO inhibited the firing rates of the recorded neurons (mean ± SEM). Paired t test, **P* < 0.05, n = 4 cells. Right, an example raw trace showing the changes in membrane potential and firing rates before, during and after application of CNO (mean ± SEM). Paired t test, **P* < 0.05, n = 4 cells.

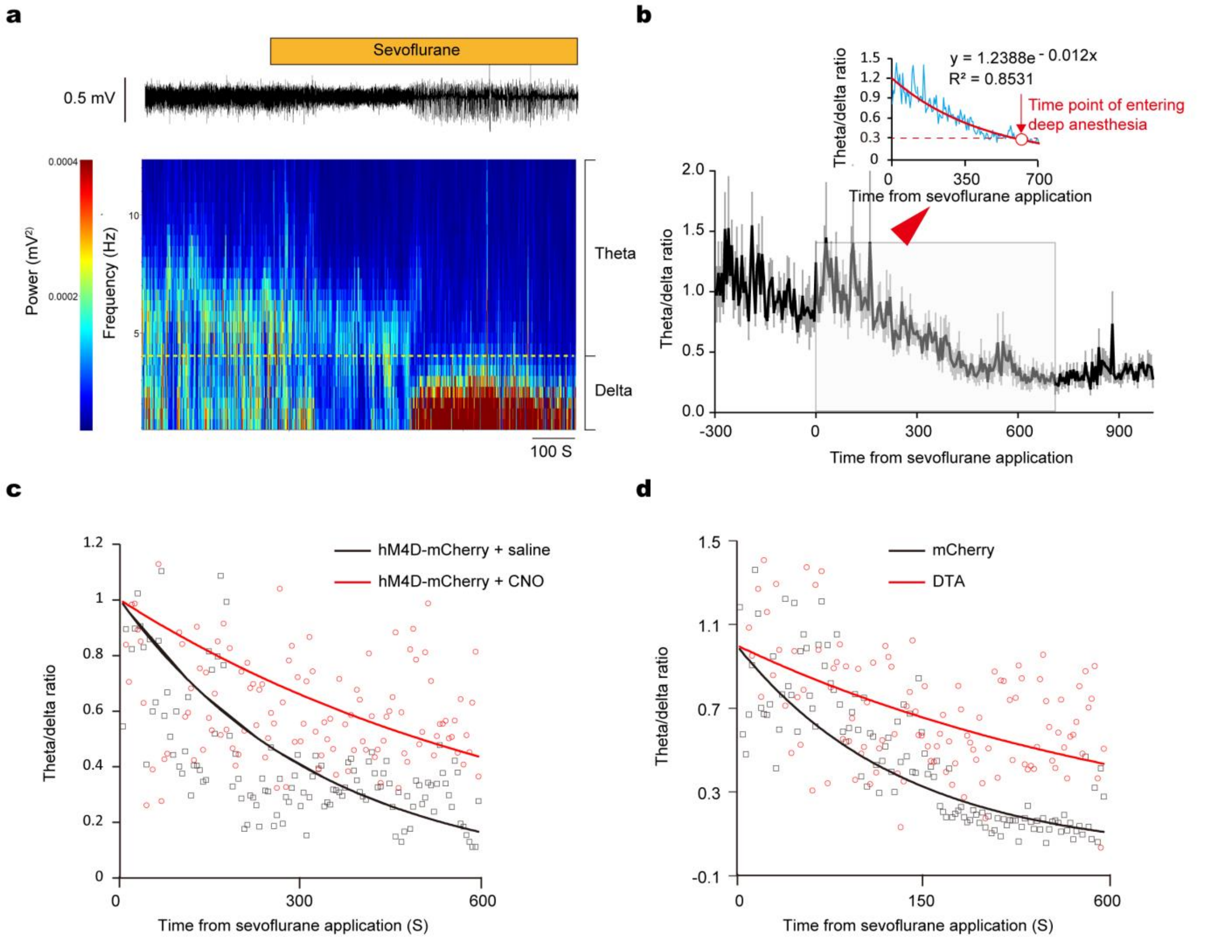

Figure S4. The determination of time to anesthesia by analyzing EEG power spectrum. a) An example EEG trace and its power spectrum before and during application of sevoflurane. b) The average ratio of EEG theta wave (4-12 Hz) to delta wave (0.5-4 Hz) power (theta/delta ratio) in the control mice (n = 8) gradually decreased after application of sevoflurane. Inset, an exponential function was used to fit to the theta/delta ratio. According to these fitting parameters, the time point where theta/delta ratio deceased to 0.3, the average theta/delta ratio of the burst suppression was selected as the time point of deep anesthesia entrance. c) The theta/delta ratio after application of sevoflurane in hM4D-mCherry mice with intraperitoneal injection of saline and CNO. d) The alterations in theta/delta ratio after application of sevoflurane after DTA lesion of EW sevoflurane-activated neurons.

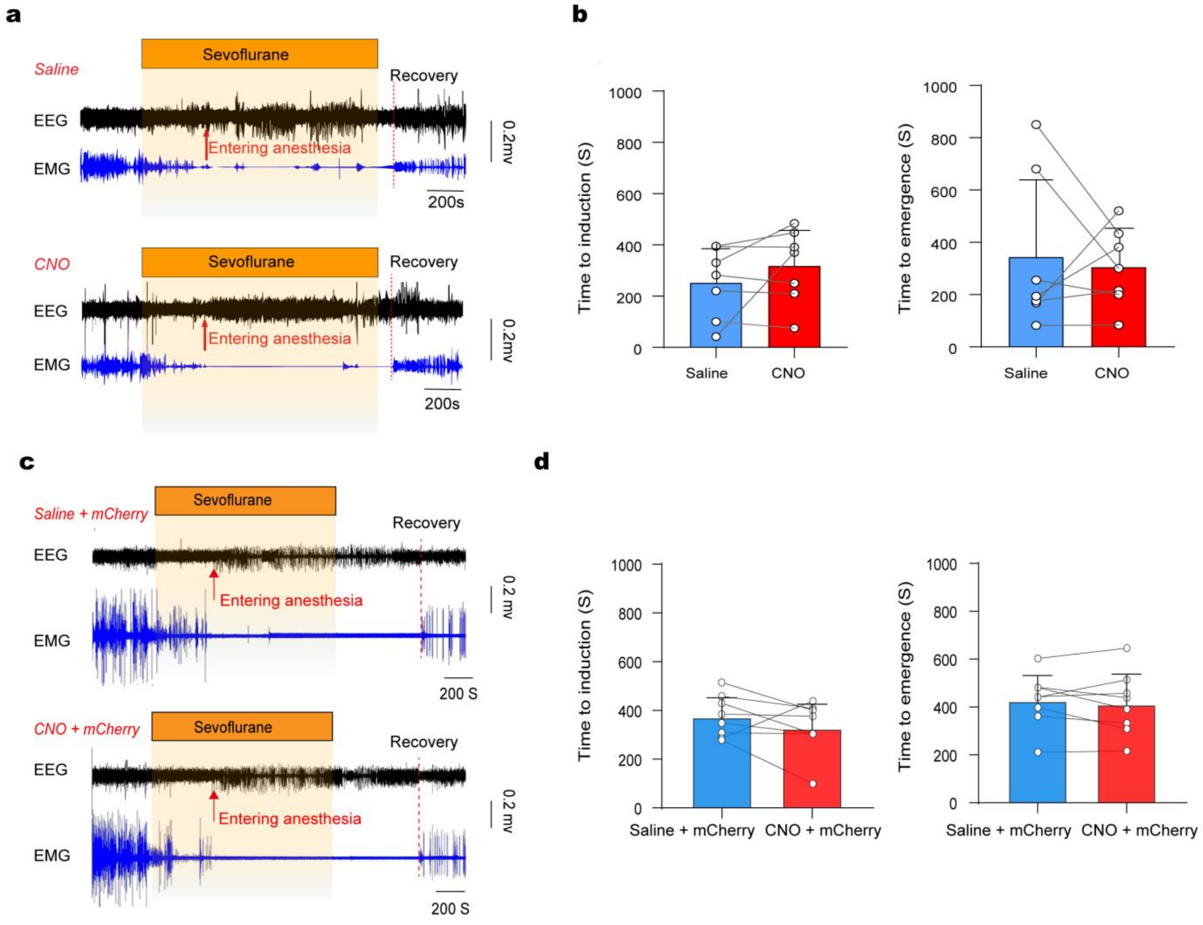

Figure S5. Sevoflurane anesthesia in control mice for chemogenetic inhibition experiment. a) Examples of EEG and EMG traces recorded before and during application of sevoflurane in wild-type mice. The arrow indicates the time point of entering anesthesia. b) Left, the time to induction in wild-type mice with intraperitoneal injection of saline and CNO (mean ± SEM). Paired t test, $P = 0.232$, n = 7 mice. Right, the time to emergence in wild-type mice with intraperitoneal injection of saline and CNO (mean ± SEM). Paired t test, $P = 0.727$, n = 7 mice. c) Examples of EEG and EMG traces recorded before and during application of sevoflurane in mice with sevoflurane-activated neurons expressing mCherry. The arrow indicates the time point of entering anesthesia. d) Left, the time to induction in mCherry-expressing mice with intraperitoneal injection of saline and CNO (mean ± SEM). Paired t test, $P = 0.232$, n = 8 mice. Right, the time to emergence in mCherry-expressing mice with intraperitoneal injection of saline and CNO (mean ± SEM). Paired t test, $P = 0.541$, n = 8 mice.

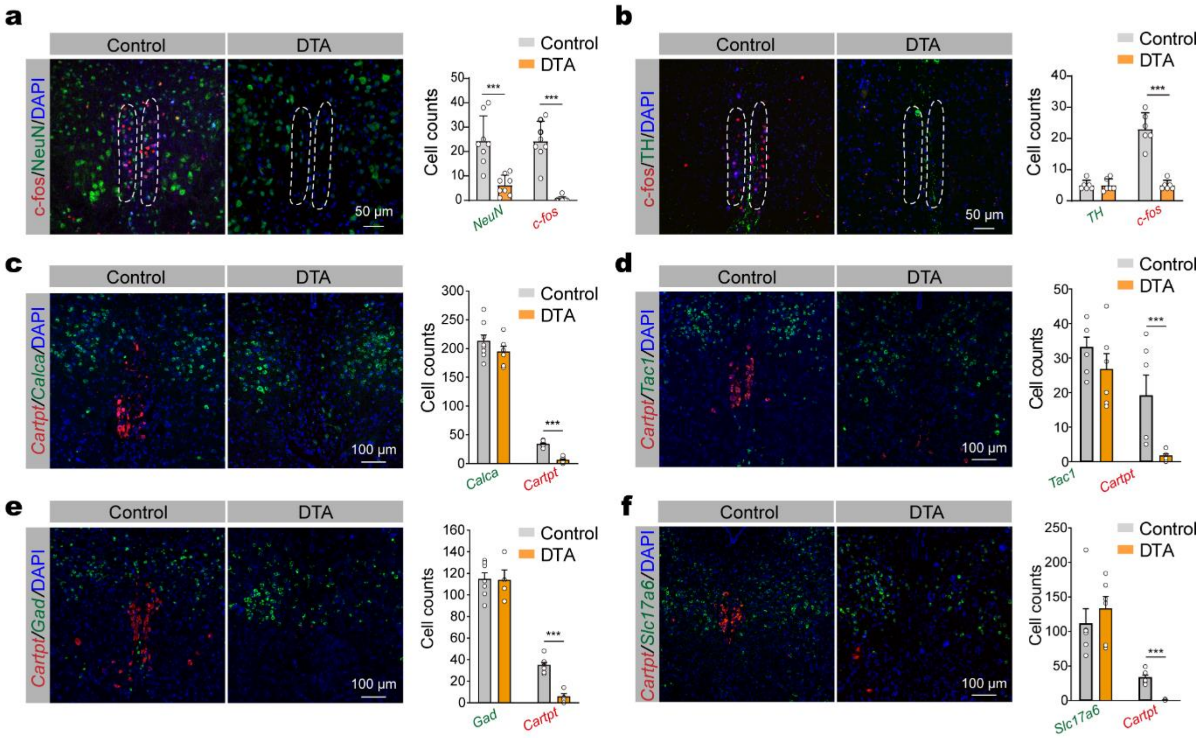

Figure S6. The validation of DTA-mediated ablation of sevoflurane-activated neurons in the EWcp. a) Left, representative immunofluorescence images of the c-fos (red), NeuN (green), and DAPI (blue). Right, histograms showing the number of NeuN$^+$ and c-fos$^+$ neurons in the midbrain EW region in control and DTA groups (mean ± SEM). Unpaired t test(NeuN) or mann whitney test(c-fos), *** $P < 0.001$, n = 8 sections from 3 mice. b) Left, representative immunofluorescence images of the c-fos (red), TH (green), and DAPI (blue). Right, histograms showing the number of TH$^+$ and c-fos$^+$ neurons in the midbrain EW region in control and DTA groups (mean ± SEM). Mann whitney test(TH) or unpaired t test(c-fos), *** $P < 0.001$, n = 6 sections from 3 mice. c) Left, representative images of two-color *in situ* hybridization between *Cartpt* (red), *Calca* (green) and DAPI (blue). Right, histograms showing the number of *Calca*$^+$ and *Cartpt*$^+$ neurons in control and DTA groups (mean ± SEM). Unpaired t test, *** $P < 0.001$, n = 6 sections from 3 mice. d) Left, representative images of two-color *in situ* hybridization between *Cartpt* (red), *Tac1* (green) and DAPI (blue). Right, histograms showing the number of *Tac1*$^+$ and *Cartpt*$^+$ neurons in control and DTA groups (mean ± SEM). Unpaired t test(*Tac1*) or wilcoxon signed rank test(*Cartpt*), *** $P < 0.001$, n = 6 sections from 3 mice. e) Left, representative images of two-color *in situ* hybridization between *Cartpt* (red), *Gad* (green) and DAPI (blue). Right, histograms showing the number of *Gad*$^+$ and *Cartpt*$^+$ neurons in control and DTA groups (mean ± SEM). Unpaired t test, *** $P < 0.001$, n = 6 sections from 3 mice. f) Left, representative images of two-color *in situ* hybridization between *Cartpt* (red), *Slc17a6* (green) and DAPI (blue). Right, histograms showing the number of *Slc17a6*$^+$ and *Cartpt*$^+$ neurons in control and DTA groups (mean ± SEM). Unpaired t test, *** $P < 0.001$, n = 6 sections from 3 mice.

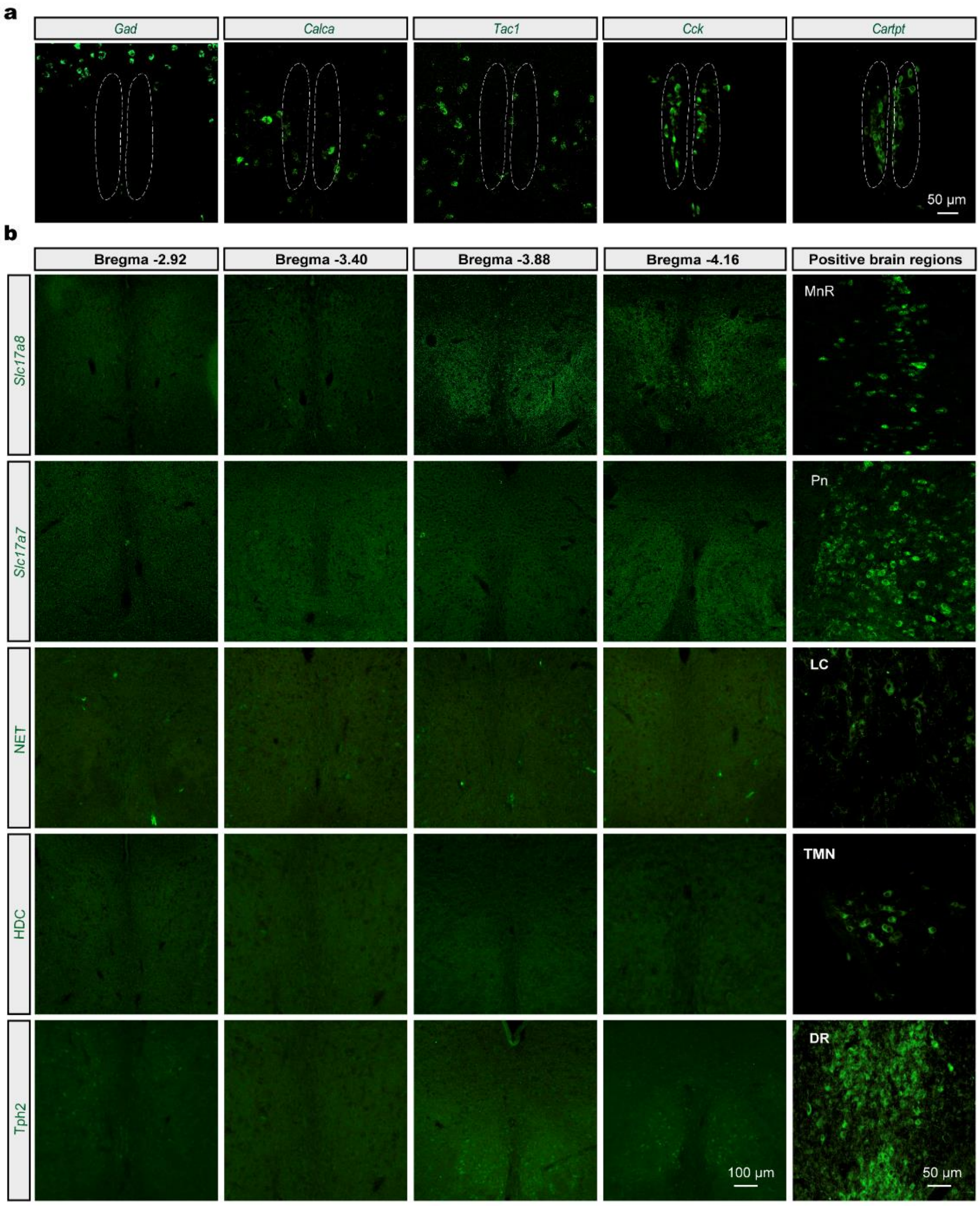

Figure S7. Cell types in the midbrain EW area. a) Representative FISH images showing positive expression of different cell type markers in the midbrain EW and the surrounding area. n = 3 mice. b) Left, representative FISH (the first two rows) and IF (the last three rows) images showing negative expression of different cell type markers in the midbrain EW and the surrounding area. Right, representative images illustrating the expression of different cell markers in the positive control brain areas. n = 3 mice.

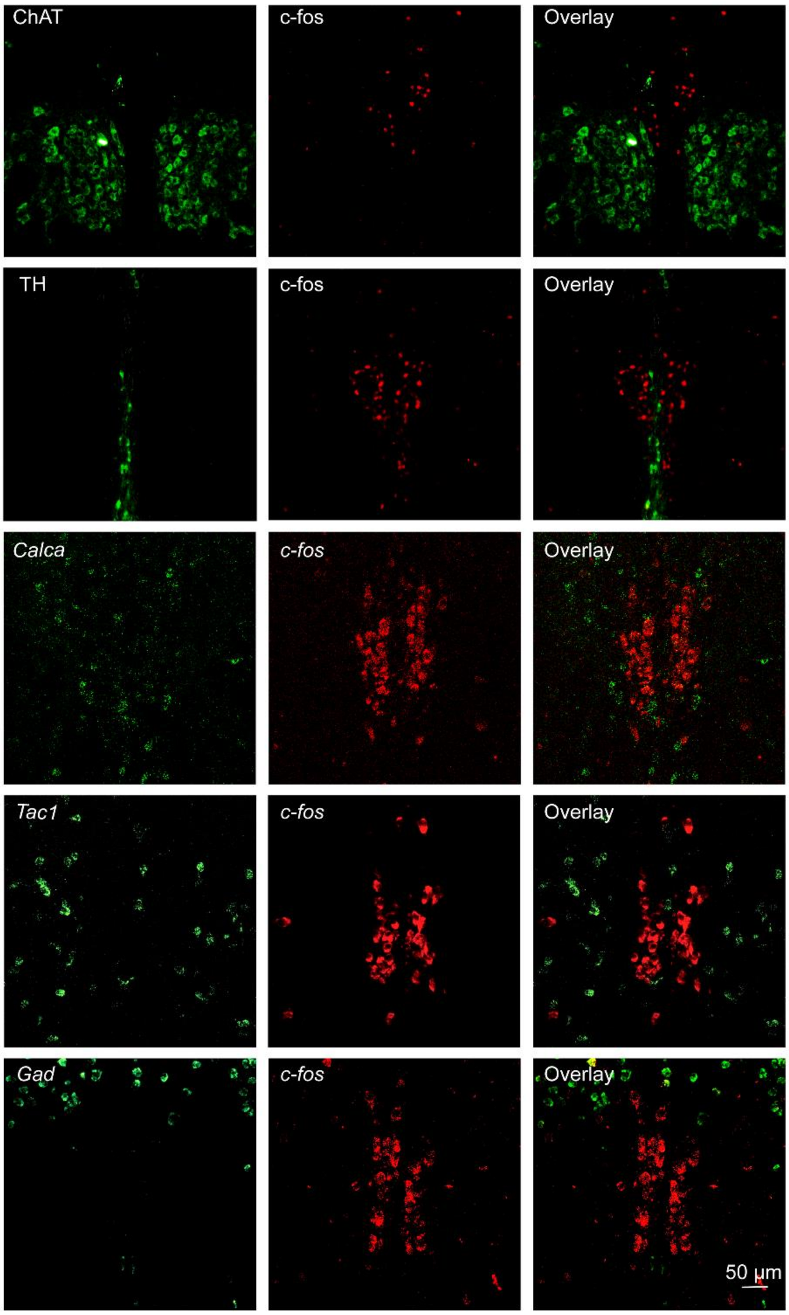

Figure S8. Sevoflurane-activated neurons are not cholinergic, dopaminergic, GABAergic, substance P$^+$ or Calca$^+$ neurons. Representative images of immunofluorescence (the first two rows) or two-color *in situ* hybridization (the last three rows) between c-fos (red) and the following markers (green): ChAT, TH, *Calca*, *Tac1*, and *Gad*. n = 3 mice.

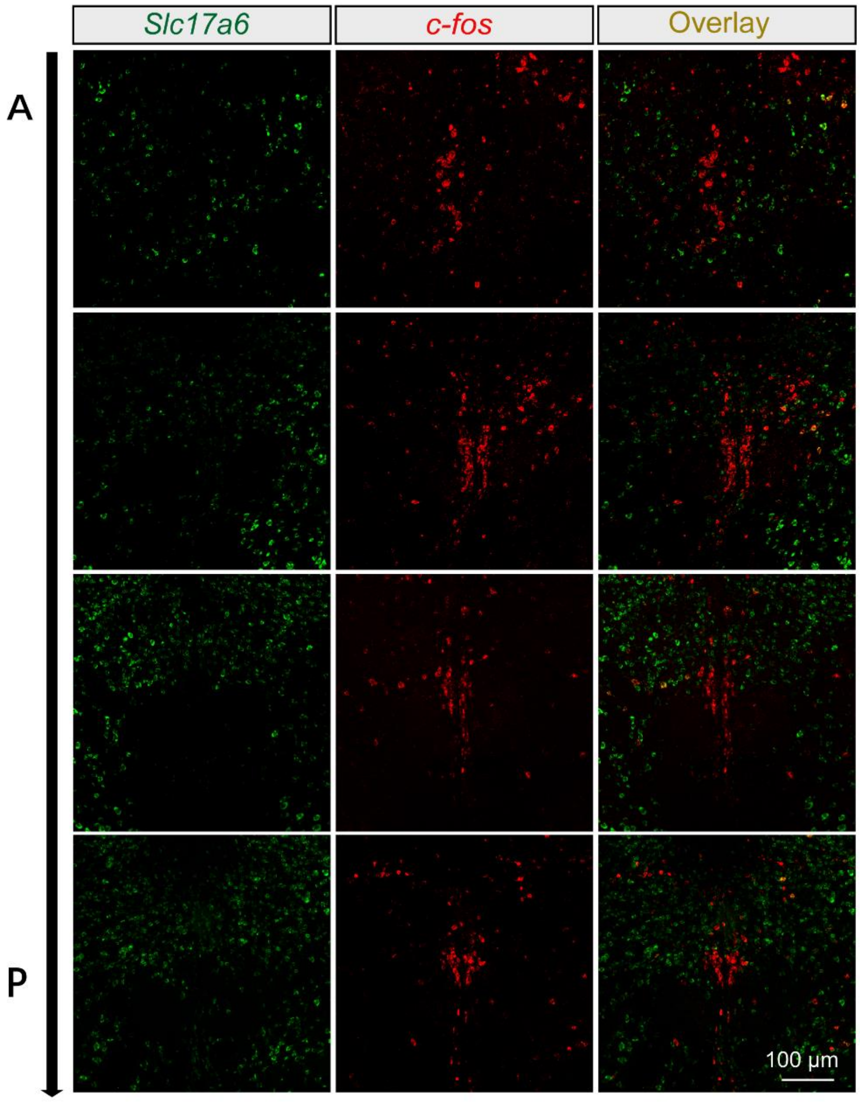

Figure S9. The majority of sevoflurane-activated neurons are non-glutamatergic neurons. Representative images of two-color *in situ* hybridization between *c-fos* (red) and *Slc17a6* (encoding VGLUT2, green) along the anterior-posterior axis. n = 3 mice. A, anterior. P, posterior.

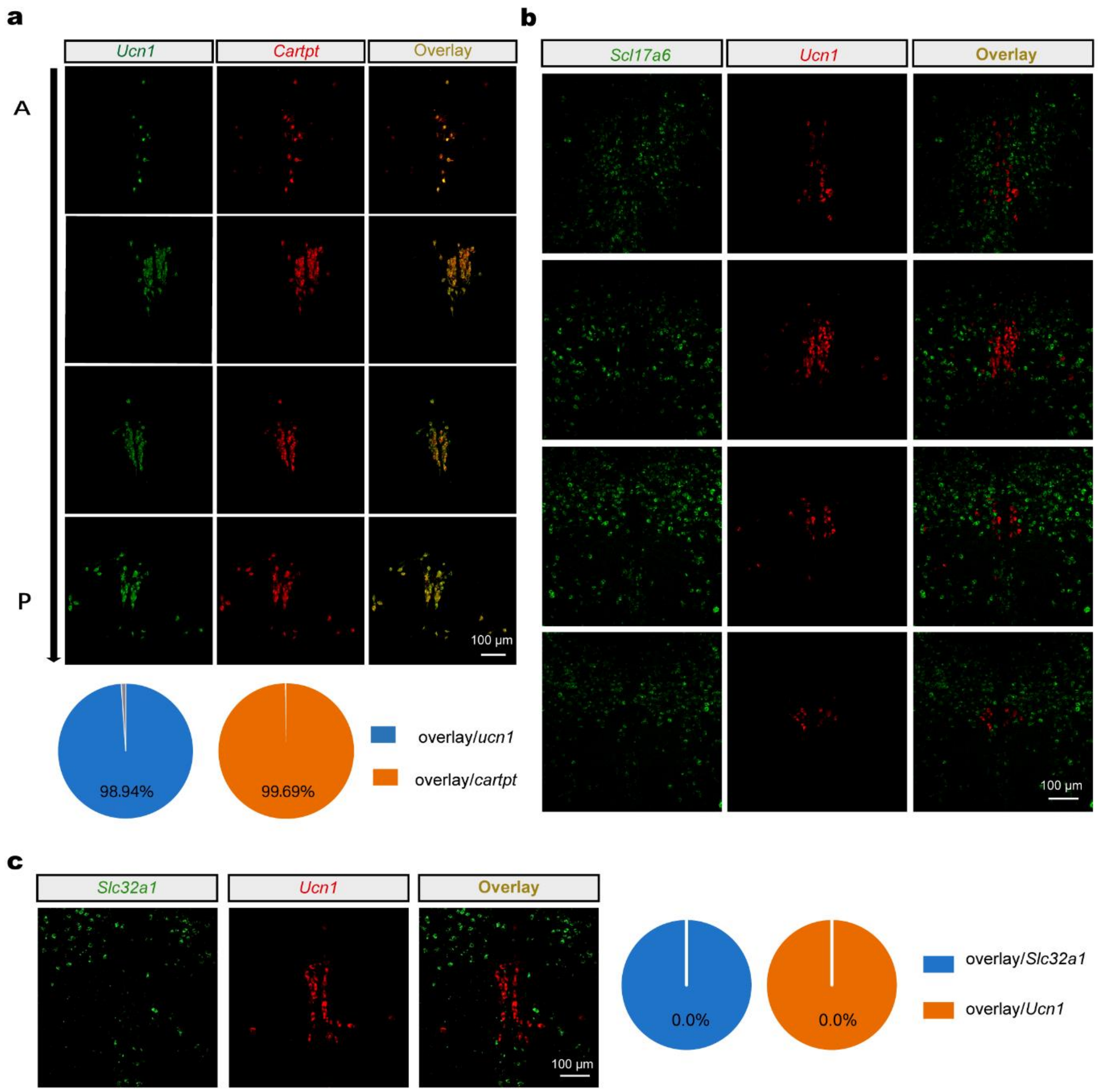

Figure S10. UCN1^{+} neurons are also positive for CART, but none of these are VGLUT2^{+}. a) Top, representative images of two-color *in situ* hybridization between *Cartpt* (red) and *Ucn1* (green) along the anterior-posterior axis. Bottom, pie chart of percentage of *Ucn1* colocalized with *Cartpt*. n = 3 mice. b) Representative images of two-color *in situ* hybridization between *Ucn1* (red) and glutamatergic marker *Slc17a6* (encoding VGLUT2) (green) along the anterior-posterior axis. n = 3 mice. A, anterior. P, posterior. c) Representative images of two-color *in situ* hybridization between *Ucn1* (red) and GABAergic marker *Slc32a1* (encoding VGAT) (green). n = 3 mice.

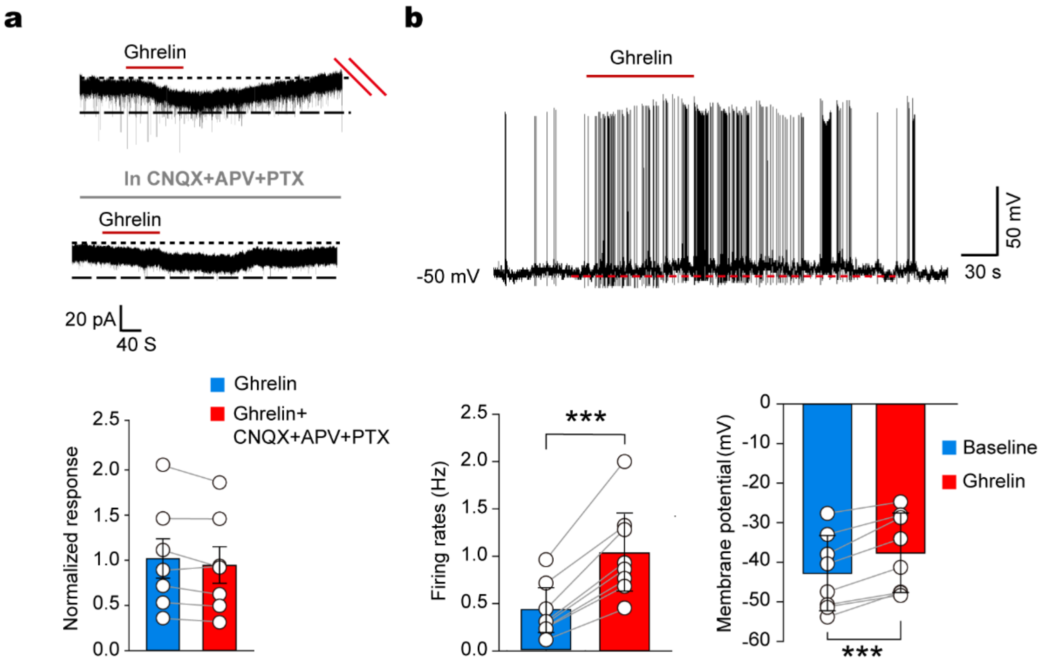

Figure S11. Ghrelin directly excites UCN1^{+} neurons. a) Effect of ghrelin on membrane currents in the presence of AMPA/NMDA glutamate receptor antagonist APV and CNQX, and GABA$_A$R antagonist picrotoxin (PTX) (mean ± SEM). Paired t test, *P* = 0.0721, n = 7 cells. b) Bath application of ghrelin caused membrane depolarization and increased the firing rates of the UCN1^{+} neurons (mean ± SEM). Firing rates: Paired t test, ****P* < 0.001, n = 8 cells. Membrane potential: Paired t test, ****P* < 0.001, n = 8 cells.

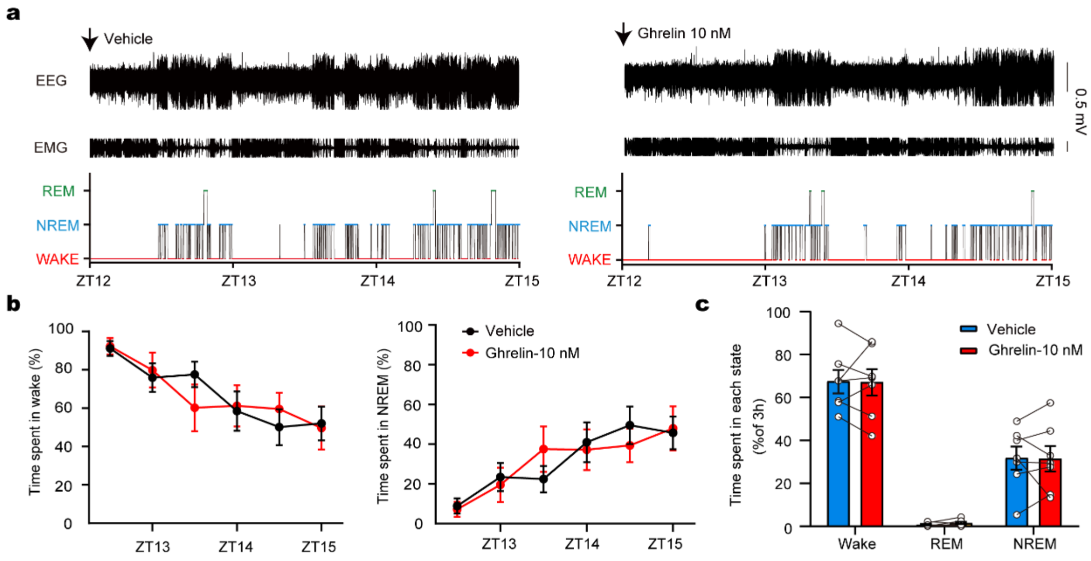

Figure S12. Local injection of ghrelin at low concentration (10 nM) in the EW region does not affect sleep. a) Representative traces of EEG, EMG and hypnogram during 3 h post-vehicle or ghrelin (10 nM) injection into midbrain EW region during the dark phase. b) Left, hourly percentage of time spent in wakefulness state post vehicle or ghrelin (10 nM) injection. Right, hourly percentage of time spent in non-rapid eye movement sleep (NREM) state during 3 h post-vehicle or ghrelin (10 nM) injection. c) Percentage of time spent in each state

during 3 h post vehicle or ghrelin (10 nM) injection (mean ± SEM). Paired t test, Wake: *P* = 0.918, REM: *P* = 0.168, NREM: *P* = 0.928, n = 7 mice.

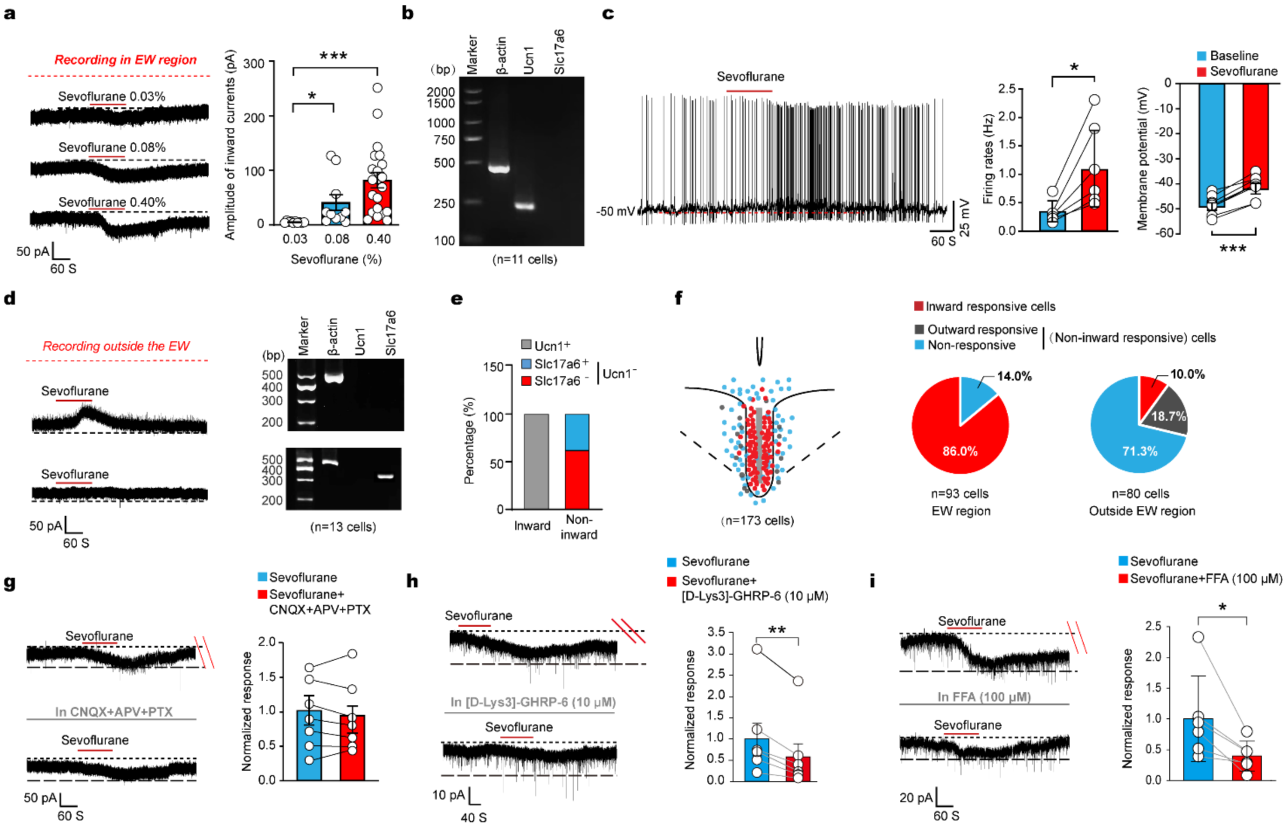

Figure S13. Sevoflurane excites UCN1^{+} neurons independently of the synaptic transmissions. a) Whole-cell patch-clamp recording of EW neurons following treatments of different concentrations of sevoflurane, noting that sevoflurane generated inward currents in a concentration-dependent manner (mean ± SEM). Kruskal-wallis one way analysis of variance on ranks, **P* < 0.05, ****P* < 0.001, 0.03%: n = 10 cells; 0.08%: n = 10 cells; 0.40%: n = 21 cells. b) Single-cell reverse transcription-polymerase chain reaction (scRT-PCR) showed that the neurons (n = 11) that exhibited inward currents in response to sevoflurane were positive for UCN1, but not VGLUT2. c) Bath application of sevoflurane increased the firing rates and caused membrane depolarization of the UCN1^{+} neurons (mean ± SEM). Firing rates: Paired t test, **P* < 0.05, n = 6 cells; membrane potential: Paired t test, ****P* < 0.001, n = 8 cells. d) scRT-PCR combined with whole-cell patch clamp showed that the neurons without inward current response in the regions outside of EW were negative for UCN1. e) Histograms showing the percentage of neurons with inward and non-inward responses expressing different markers. f) The spatial distribution of cells with different responses, noting that cells with inward current response were mainly distributed in the midbrain EW region (light zone) activated by sevoflurane. On the contrary, neurons without inward response were mainly distributed outside the EW region (dark zone). g) Effects of sevoflurane on the membrane currents in the presence of AMPA/NMDA glutamate receptor antagonist APV and CNQX, and the $GABA_AR$ antagonist picrotoxin (PTX) (mean ± SEM). Paired t test, *P* = 0.8523, n = 7 cells. h) Left, an exemplar raw trace showing the effects of sevoflurane on the membrane currents in the presence of GHSR antagonist. Right, histograms summarizing the changes of sevoflurane-induced inward currents before and after blocking GHSR

(mean ± SEM). Paired t test, ***P* < 0.01, n = 7 cells. i) Left, an exemplar raw trace showing the effects of sevoflurane on the membrane currents in the presence of NSCC antagonist (100 μM). Right, histograms summarizing the changes of sevoflurane-induced inward currents before and after blocking NSCCs (mean ± SEM). Paired mann-whitney rank sum test, **P* < 0.05, n = 6 cells.

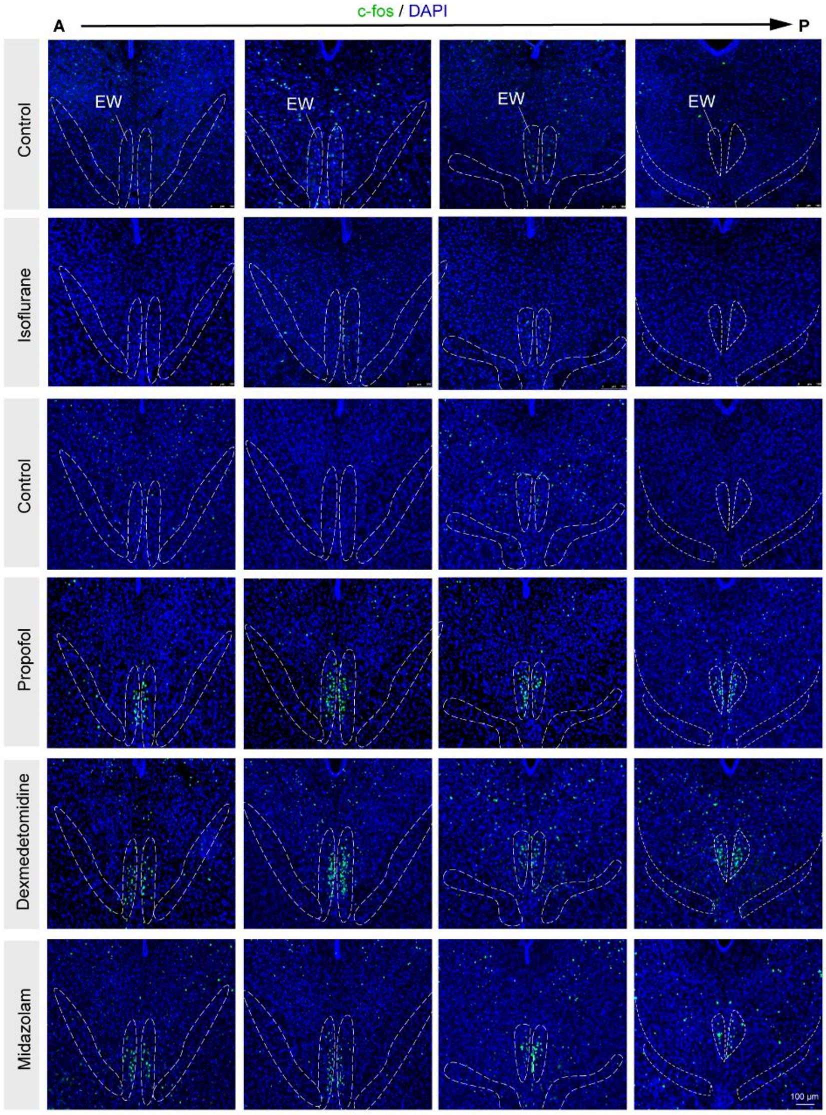

Figure S14. Effects of the isoflurane, propofol, dexmedetomidine and midazolam on the activities of the sevoflurane-activated neurons in the EW region. Representative immunofluorescence images showing c-fos expression in the EW region after anesthesia induced by isoflurane (1.2%), propofol (180 mg/kg), dexmedetomidine (100 μg/kg), midazolam (50 mg/kg) and control condition with either vehicle or oxygen exposure alone.

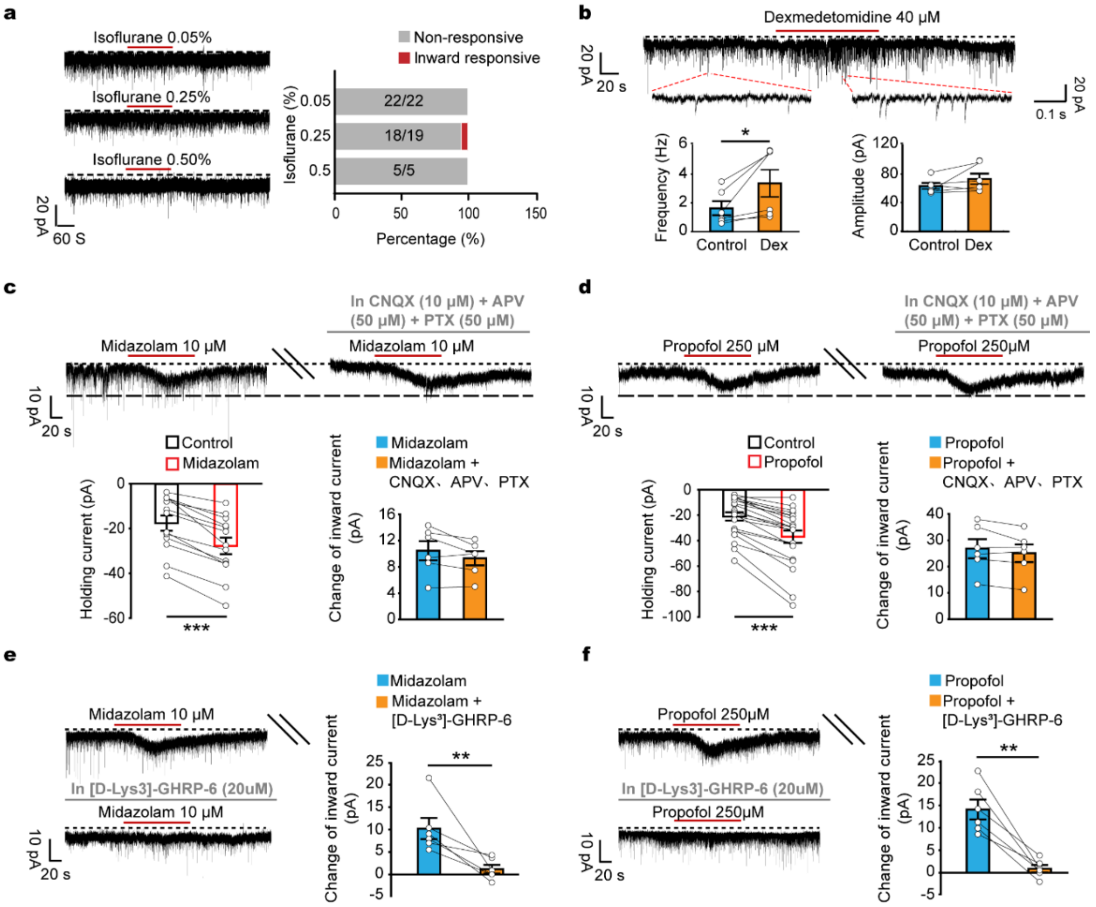

Figure S15. The effects of the isoflurane, dexmedetomidine, midazolam and propofol on the activities of the UCN1$^+$ neurons. a) Whole-cell patch-clamp recording of EW neurons following treatments of different concentrations of isoflurane. b) Effects of dexmedetomidine on the membrane currents. Frequency: Paired t test, **P* < 0.05, n = 6 cells; amplitude: Paired t test, *P* = 0.172, n = 6 cells. c) and d) Effects of midazolam (c) and propofol (d) on the membrane currents in the absence and presence of AMPA/NMDA glutamate receptor antagonist APV and CNQX, and the $GABA_AR$ antagonist PTX. Midazolam, holding current: Paired t test, ****P* < 0.001, n = 13 cells; change in inward current: Paired t test, *P* = 0.123. Propofol, holding current: Paired t test, ****P* < 0.001, n = 21 cells; change in inward current: Paired t test, P = 0.126. e and f) Effects of midazolam and propofol on the membrane currents in the presence of GHSR antagonist. Midazolam: Paired t test, ***P* < 0.01, n = 6 cells; propofol: Paired t test, ***P* < 0.01, n = 6 cells.
